# Obesity-linked circular RNA circTshz2-2 regulates the neuronal cell cycle and spatial memory in the brain

**DOI:** 10.1038/s41380-021-01303-x

**Published:** 2021-09-24

**Authors:** Gwangho Yoon, Yeong-Hwan Lim, Danbi Jo, Juhee Ryu, Juhyun Song, Young-Kook Kim

**Affiliations:** 1 BioMedical Sciences Graduate Program (BMSGP), Chonnam National University, Hwasun, Jeollanam-do 58128 Republic of Korea; 2 Department of Biochemistry, Chonnam National University Medical School, Hwasun, Jeollanam-do 58128 Republic of Korea; 3 Department of Anatomy, Chonnam National University Medical School, Hwasun, Jeollanam-do 58128 Republic of Korea; 4 College of Pharmacy and Research Institute of Pharmaceutical Sciences, Kyungpook National University, Buk-gu, Daegu, 41566 Republic of Korea

**Keywords:** Molecular biology, Neuroscience, Cell biology

## Abstract

Metabolic syndromes, including obesity, cause neuropathophysiological changes in the brain, resulting in cognitive deficits. Only a few studies explored the contribution of non-coding genes in these pathophysiologies. Recently, we identified obesity-linked circular RNAs (circRNA) by analyzing the brain cortices of high-fat-fed obese mice. In this study, we scrutinized a conserved and neuron-specific circRNA, circTshz2-2, which affects neuronal cell cycle and spatial memory in the brain. Transcriptomic and cellular analysis indicated that circTshz2-2 dysregulation altered the expression of cell division-related genes and induced cell cycle arrest at the G2/M phase of the neuron. We found that circTshz2-2 bound to the YY1 transcriptional complex and suppressed *Bdnf* transcription. Suppression of circTshz2-2 increased BDNF expression and reduced G2/M checkpoint proteins such as Cyclin B2 and CDK1 through BDNF/TrkB signaling pathway, resulting in cell cycle arrest and neurite elongation. Inversely, overexpression of circTshz2-2 decreased BDNF expression, induced cell cycle proteins, and shortened the neurite length, indicating that circTshz2-2 regulates neuronal cell cycle and structure. Finally, we showed that circTshz2-2 affects spatial memory in wild-type and obese mice. Our data have revealed potential regulatory roles of obesity-related circTshz2-2 on the neuronal cell cycle and memory function providing a novel link between metabolic syndromes and cognitive deficits.

## Introduction

Countless people suffer from various diseases related to metabolic imbalances including obesity, diabetes mellitus, and hypertension [1]. Metabolic imbalances are manifested by eating a diet high in sugar, fat, and cholesterol which results in the malfunction of the liver, pancreas, muscle, and adipocytes [2]. Numerous signaling pathways related to both glucose and lipid metabolism are dysregulated in metabolic syndromes, leading to an imbalance in the glucose, free fatty acid, and cholesterol levels in the blood and various organs [3]. The insulin balance can also be affected resulting in significant irregularities in the glucose metabolism [4]. Recent studies have shown that metabolic imbalances, which have been believed to affect only the peripheral organs, destroy brain function [5]. Increased local and systemic inflammation following metabolic imbalance causes blood-brain barrier collapse and increases the infiltration of glucose, insulin, free fatty acid, and cholesterol into the brain [6]. The imbalance of these factors manifests as insulin resistance and accelerates inflammatory responses in glial and neuronal cells, resulting in impaired synaptic plasticity, neuronal cell damage, and memory impairment [7]. Patients with obesity and diabetes mellitus present with diverse symptoms including impaired memory consolidation and cognitive decline at later stages of disease progression [8]. However, there are relatively few studies describing the mechanism between metabolic imbalance and cognitive decline. In addition, the identification of the exact regulatory factors controlling these outcomes is not known making the development of effective therapeutics for treating metabolic imbalance-related cognitive deficits difficult.

For decades, most studies investigated to understand brain function and its disorders have focused on identifying the role of various proteins [9]. Although these findings provide critical information regarding the roles of various proteins in the brain, it is still insufficient to comprehensively explain brain physiology and pathology. Recent studies have shed new light on the regulatory roles of circular RNA (circRNA) in brain function [10]. CircRNA is a single-stranded RNA that is covalently closed at the 5′ and 3′ termini of the exons produced by a novel process called back-splicing [11]. The primary function of circRNAs has been described as the regulation of gene expression via microRNA sponging or RNA-binding protein sequestration [12]. Some circRNAs are translated into functional peptides which act as tumor-suppressors in various cancers [13]. The number and diversity of the circRNAs are significantly enriched in the brain when compared to other organs, suggesting that these transcripts may play critical roles in neuronal development and differentiation, synaptic plasticity, and memory formation [14]. However, few studies have been described the role(s) of circRNAs in the development, differentiation, and progression of brain disease [15, 16].

A handful of studies have described the role of circRNAs in obesity-related conditions [17]. CircSAMD4A was reported as an adipogenesis regulator in obese patients [18] which acts to inhibit preadipocyte differentiation by binding to miR-138-5p and regulating *EZH2* expression. Moreover, it was observed that the expression of ciRS-7/CDR1as, which is one of the most famous circRNAs, decreased in both obesity and diabetes-related transgenic mouse models [19]. CircHIPK3 has been described as a regulator of obesity-induced insulin resistance and is known to bind miR-192-5p and upregulate *FOXO1* gene expression [20]. CircARF3 is linked to obesity-induced inflammation in mouse adipose tissues [21] where it functions as a miR-103 sponge and accelerates inflammation by regulating TNF receptor-associated factor 3 (*TRAF3*) expression. However, no study has described the roles of these obesity-related circRNAs in neuronal differentiation, development, and brain function.

Recently, we reported the unique expression patterns of circRNAs in the brain cortices of high-fat-fed obese mice [22]. In this study, we specify the role of circTshz2-2, which is highly expressed in the brain cortices of obese mice, in neural structure change, neuronal cell cycle regulation, and spatial memory improvement.

## Materials and methods

Animal care, cell line culture, primary culture, transfection, siRNA design, cloning of circRNA, cell fractionation, RNA-sequencing (RNA-seq), bioinformatic analysis, RNA isolation and PCR, western blot, neurite length and Sholl analysis, cell cycle analysis, immunofluorescence, RNA-binding protein immunoprecipitation (RNA-IP), osmotic pump implantation, T-maze spontaneous alternation test, and statistical analysis were performed as described in the Extended Data Supplementary materials and methods section.

## Results

### Identification of obesity-related circRNAs specifically expressed in the neurons

To investigate the roles of obesity-related circRNAs in neuronal function, we identified those circRNAs that are specifically expressed in Neuro-2A neuronal origin cells rather than in BV-2 microglial or C8-D1a astrocytic cells (Supplementary Fig. S1). We screened 15 circRNAs, of which nine were specifically expressed in Neuro-2A cells; circTtc3, circZbtb20, circSatb1, circTshz2-1, circTshz2-2, circMapk4, circSobp, circRims2-1, and circRims2-2. Only circSorl1 was found to be uniquely expressed in BV-2 microglial cells. The rest of the circRNAs were primarily expressed in C8-D1a astrocytic cells; circMpped1, circP1ekha5-1, circArhgap5, circUbe2k, and circTmem44. Thus, these obesity-related circRNAs show differential expression in the various cell types that make up the brain. We assume that these circRNAs will perform some unique functions in each cell type. In addition, nine of the 15 circRNAs are specifically expressed in the neuron, suggesting that these obesity-related circRNAs play some critical roles in neuronal function.

### The expression of circTshz2-1 and circTshz2-2 is markedly increased in the differentiated neuron

We then tried to select a subset of these neuron-specific circRNAs that undergo differential expression in response to retinoic acid-induced differentiation in Neuro-2A cells. The expression of genes related to differentiation (*Rbfox3*, *Chat*, *Map2*) and synaptic vesicle maturation (*Stx1a* and *Syp*) were used as markers of neuronal differentiation. The expression of these marker genes was higher in differentiated cells on day 3 than undifferentiated cells on day 1 (Supplementary Fig. S2). In addition, the expression of several circRNAs steadily increased over the 3 days of neuronal differentiation; circTshz2-1, circTshz2-2, circSatb1, circTtc3, and circRims2-1 (Supplementary Fig. S3A). Among the host genes of these circRNAs, only the expression of *Tshz2* and *Satb1* increased during differentiation, and these increases mirrored the patterns of expression in their respective circRNAs. The host gene for circTtc3 was not detected in any of the cells evaluated in these assays and on the evaluated circRNAs only circTshz2-1 and circTshz2-2 showed further increases in their expression after 3 days of differentiation (Supplementary Fig. S3B). Thus, we selected these circRNAs for further study.

### Characterization and conservation of circTshz2-1 and circTshz2-2

A few studies have reported functions for both circTshz2-2 and Tshz2. It was reported that circTshz2-2 increases adipogenesis-related gene expression, such as *Pparγ* and *Fabp4*, in obesity [23] while other reports suggest that Tshz2 regulates tumorigenesis and epigenetic methylation in breast and prostate cancers, and is involved in the development of zebrafish [24, 25]. However, neither circTshz2-2 nor Tshz2 has been characterized in the brain. To characterize the tissue distribution of Tshz2 expression, we examined the publicly available human GTEx RNA-seq data [26]. Human TSHZ2 is expressed throughout all tissues (Supplementary Fig. S4) and is widely expressed throughout the cortex, hypothalamus, and spinal cord, indicating that it also has a significant function in the brain.

**Fig. 1:**
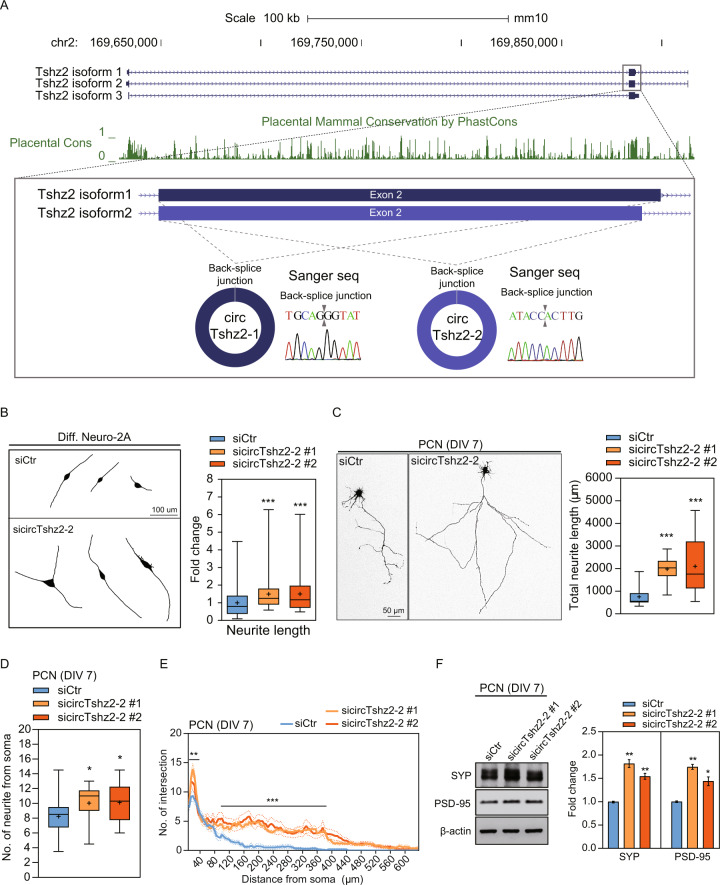
Regulation of neuronal structures by circTshz2-2. **A** Genomic information and conservation of circTshz2-2 in mouse *Tshz2* locus (mm10). Genomic information and PhastCons data were obtained from the USCS Genome Browser. Back-splice junctions of circRNAs identified by Sanger sequencing are indicated as a silver triangle. **B** Changes in neural structures and neurite length following circTshz2-2 knockdown in differentiated (Diff.) Neuro-2A cells at day 5. siCtr denotes the negative control siRNA. **C** Changes in neural structures and total neurite length following circTshz2-2 knockdown in mouse primary cortical neurons (PCN) after 7 days in vitro (DIV 7). **D** Changes in the number of neurites from each soma following circTshz2-2 knockdown in PCN at DIV 7. The whiskers in graphs **B**–**D** represent the minimum and maximum values while the box line represents the first quartile, median, and third quartile, respectively. The “+“ indicates the mean. **E** Changes in neural complexity following circTshz2-2 knockdown in PCN at DIV 7. The number of intersections was analyzed using the Sholl analysis method as described in Fig. S9E and results are described as the mean (solid line) ± SEM (dot line). **F** Changes in SYP and PSD-95 protein levels following circTshz2-2 knockdown in PCN at DIV 7. Data are represented as the mean ± SEM (*n* = 3). Structural data for **B**–**E** represents the findings from at least three independent experiments (*n* = 3) with more than ten neurons analyzed per replicate. Statistical significance for **B**–**F** was determined using an unpaired two-tailed *t-*test with Welch’s correction. The data in **E** were evaluated using an ordinary two-way ANOVA test. *p < 0.05, **p < 0.01, ***p < 0.001.

We went on the evaluate the various isoforms of *Tshz2* in the neuron and used this information to confirm the genomic locus and origin of the circTshz2-1 and circTshz2-2 transcripts. The UCSC Genome Browser identifies three Tshz2 isoforms in mice; two transcripts with three exons including either longer exon 2 (isoform 1) or shorter exon 2 (isoform 2), and a transcript with two exons (isoform 3) (Fig. 1A) [27]. In addition, our previous RNA-seq data identified two isoforms of circTshz2; circTshz2-1 and circTshz2-2. Using Sanger sequencing analysis, we found that circTshz2-1 and circTshz2-2 were produced from the back-splicing of exon 2 in Tshz2 isoform 1 or 2, respectively (Fig. 1A). We designed two sets of primers to check the expression of *Tshz2* isoforms 1 and 2 and detected transcripts of the appropriate size for both (Supplementary Fig. S5A, B). We observed that the expression of *Tshz2* isoforms 1 and 2 also increased in differentiated neurons showing a similar expression pattern to their corresponding circRNAs (Supplementary Fig. S5C).

Transcript conservation in placental mammals was evaluated using PhastCons and PhyloP which showed that the exon 2 isoforms from *Tshz2* producing circTshz2-1 and circTshz2-2 are well preserved (>96%) across a broad range of species (Fig. 1A and Supplementary Fig. S6). This was then confirmed in the expression evaluations for both circTshz2-1 and circTshz2-2 in the rat primary cortical neurons (Supplementary Fig. S7A) and the human SH-SY5Y neuronal cells (Supplementary Fig. S7B). We also confirmed that circTshz2-2 expression increased in mature rat primary cortical neurons and differentiated SH-SY5Y cells in a day-dependent manner (Supplementary Fig. S7A, B). Additionally, from the public GEO dataset, we found that circTshz2-2 expression also increased during the embryonic development of the hippocampus and adult brain development (Supplementary Fig. S7C). However, the expression of circTshz2-1 showed no change during the differentiation of rat cortical neurons (Supplementary Fig. S7A). These results suggest that the expression pattern and function of circTshz2-2 are better preserved than those of circTshz2-1 in neural tissues from different species.

Next, we isolated the nuclear and cytoplasmic fractions from both undifferentiated and differentiated Neuro-2A cells in order to evaluate the intracellular localization of circTshz2-1, circTshz2-2, and Tshz2. Both circTshz2-1 and circTshz2-2 were slightly more prevalent in the cytoplasmic fraction of both the undifferentiated and differentiated Neuro-2A cells (Supplementary Fig. S8). All isoforms of Tshz2 were more prevalent in the nucleus than in the cytoplasm. These results suggest that circTshz2-1 and circTshz2-2 might be involved in controlling neuronal functions in both the nucleus and the cytoplasm. Given these results, we decided to examine the role of circTshz2-2 as this showed the highest degree of expression conservation in the neurons.

### CircTshz2-2 regulates neurite elongation and neural complexity

To examine the role of circTshz2-2 in neuronal function, we designed two siRNA spanning the back-splicing junction of circTshz2-2, which contained at least seven base pairs from the 5′ and 3′ termini of exon 2, respectively (Supplementary Fig. S9A). Differentiated Neuro-2A cells were transfected with each of these independent siRNAs and were shown to experience a significant degree of circTshz2-2 downregulation (~80%) without affecting the expression of circTshz2-1 and the *Tshz2* host gene (Supplementary Fig. S9B). Inhibition of circTshz2-2 expression enhanced neurite length in differentiated cells compared to the control siRNA-treated cells without affecting either the number of neurites from the soma or the degree of secondary branching in these samples (Fig. 1B and Supplementary Fig. S9C). We also constructed an overexpression vector to produce circTshz2-2. Differentiated cells were transfected with this plasmid and a significant increase of circTshz2-2 expression was confirmed (Supplementary Fig. S9D). We observed significantly shorter neurites in circTshz2-2 overexpressed differentiated cells than in the control vector-treated cells (Supplementary Fig. S9E). Therefore, these results indicate that circTshz2-2 regulates neurite elongation.

Neuronal cell lines, even differentiated ones, experience some limitations in analyzing neurite complexity, including a limit in the number of neurites from the soma and secondary branching in these cells [28]. To examine the changes in natural neurite complexity and network following the circTshz2-2 knockdown, mouse primary cortical neurons were cultured and transfected with two independent siRNAs and a GFP plasmid. We observed significant suppression (~60%) of circTshz2-2 without any changes in circTshz2-1 or *Tshz2* host gene expression in these cells (Supplementary Fig. S9F). CircTshz2-2 suppression promoted neurite elongation approximately two-fold compared to the negative control in mouse primary cortical neurons (Fig. 1C). The primary cortical neurons were then reconstructed and analyzed for neurite complexity using the Sholl analysis method (Supplementary Fig. S9G). CircTshz2-2 knockdown significantly increased the number of neurites from the soma (Fig. 1D) and the number of intersections (Fig. 1E) in mouse primary cortical neurons. The intersections between the concentric rings and neurites in the Sholl analysis were also significantly increased at a 5- to 35-micrometer distance from the soma. There were also marked increases in the intersections at a 100 to 380-micrometer distance from the soma, indicating that the inhibition of circTshz2-2 expression enhanced neurite elongation and complexity in these cells. In addition, we also observed that the proteins related to the pre-synapse (Synaptophysin, SYP) and post-synapse (postsynaptic density protein 95 kDa, PSD-95), which are measures of synapse formation, were all increased following circTshz2-2 knockdown in primary cortical neurons compared to the negative control (Fig. 1F). This suggests that circTshz2-2 regulates neuronal network complexity by increasing the expression of the synaptic proteins. Taken together, these results suggest that circTshz2-2 regulates neurite elongation and complexity in the neuron.

### CircTshz2-2 alters the expression of genes related to cell cycle and neuronal function

**Fig. 2:**
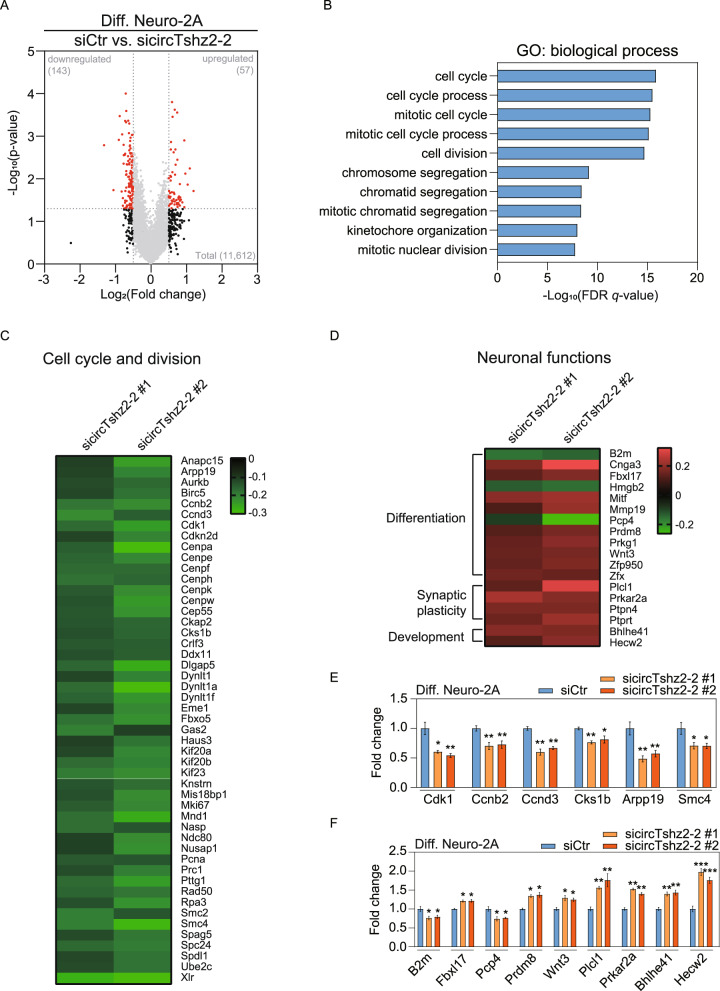
Transcriptional response to circTshz2-2 dysregulation. **A** The genes experiencing significant differential expression in response to circTshz2-2 knockdown in differentiated (Diff.) Neuro-2A cells at day 5. Those genes with a *p* value of <0.05 and those with an expression change of more than 50% in the circTshz2-2-depleted cells compared to negative control are denoted by a red dot. siCtr indicates the negative control siRNA. **B** The Gene Ontology (GO) analysis of the 169 differentially expressed protein-coding genes in these datasets. The top 10 GO terms based on the false discovery rate (FDR) *q* value are shown. **C** The expression of genes related to cell cycle and cell division following the circTshz2-2 knockdown. **D** The expression of genes related to neuronal function following the circTshz2-2 knockdown. In **C** and **D**, the color bars represent the difference in expression compared to the negative control siRNA. **E** The expression changes in the genes involved in cell cycle regulation and chromosome segmentation following circTshz2-2 knockdown were confirmed in the differentiated Neuro-2A cells at day 5 and depicted as the mean ± SEM (*n* = 3). **F** The expression changes in genes involved in neuronal function following circTshz2-2 knockdown were confirmed in the differentiated Neuro-2A cells at day 5 and depicted as the mean ± SEM (*n* = 3). In **A**, and **C**–**F**, the statistical significance was analyzed using an unpaired two-tailed *t-*test with Welch’s correction; *p < 0.05, **p < 0.01, ***p < 0.001.

We analyzed transcriptomic changes in response to changes in circTshz2-2 expression using RNA-seq to gain more insight into the molecular mechanism underlying circTshz2-2 function in differentiated Neuro-2A cells. To select statistically significant genes altered by two independent circTshz2-2 siRNAs, the transcriptome was analyzed using two independent algorithms (see “Methods”). We identified 57 upregulated and 143 downregulated genes following circTshz2-2 inhibition (Fig. 2A). Among these genes, 169 were shown to be protein-coding genes (Supplementary Fig. S10) and these were then evaluated using gene ontology (GO) to identify any common functional pathways. Strikingly, the top 10 GO terms in the biological function were entirely related to the cell cycle and division process (Fig. 2B), suggesting that the primary function of circTshz2-2 may be related to the regulation of the cell cycle. All the genes belonging to the cell cycle processes were significantly downregulated following circTshz2-2 knockdown in the RNA-seq dataset (Fig. 2C). In addition to the genes involved in the cell cycle, we found that some dysregulated genes were related to neural functions such as differentiation, development, and synaptic plasticity (Fig. 2D), indicating circTshz2-2 plays an important role in the regulation of various neuronal functions. We then used qRT-PCR analysis to verify the downregulation of the genes related to both the cell cycle checkpoints (*Cdk1*, *Ccnb2*, *Ccnb3*, *Cks1b*) and chromosome segregation (*Arpp19* and *Smc4*) in circTshz2-2-depleted cells (Fig. 2E). We also confirmed the change in the expression patterns of the genes related to differentiation (*B2m*, *Fbxl17*, *Pcp4*, *Prdm8*, *Wnt3*), development (*Bhlhe41* and *Hecw2*), and synaptic plasticity (*Plcl1* and *Prkar2a*) identified in the RNA-seq analyses (Fig. 2F). We again measured the expression of genes belonging to cell cycle checkpoints (*Cdk1*, *Ccnb2*, *Ccnd3*, *Cks1b*), chromosome segregation (*Arpp19*, *Smc4*), and neuronal function (*B2m*, *Fbxl17*, *Plcl1*, *Hecw2*) in differentiated cells after circTshz2-2 overexpression. These cells showed the opposite pattern of expression to those genes in circTshz2-2-depleted differentiated cells (Supplementary Fig. S11), indicating that circTshz2-2 may affect neurite elongation and complexity by regulating the genes associated with both the cell cycle and neuronal function.

### Downregulation of circTshz2-2 increases polyploidy via G2/M arrest and kinetochore malfunction in the neuron

**Fig. 3:**
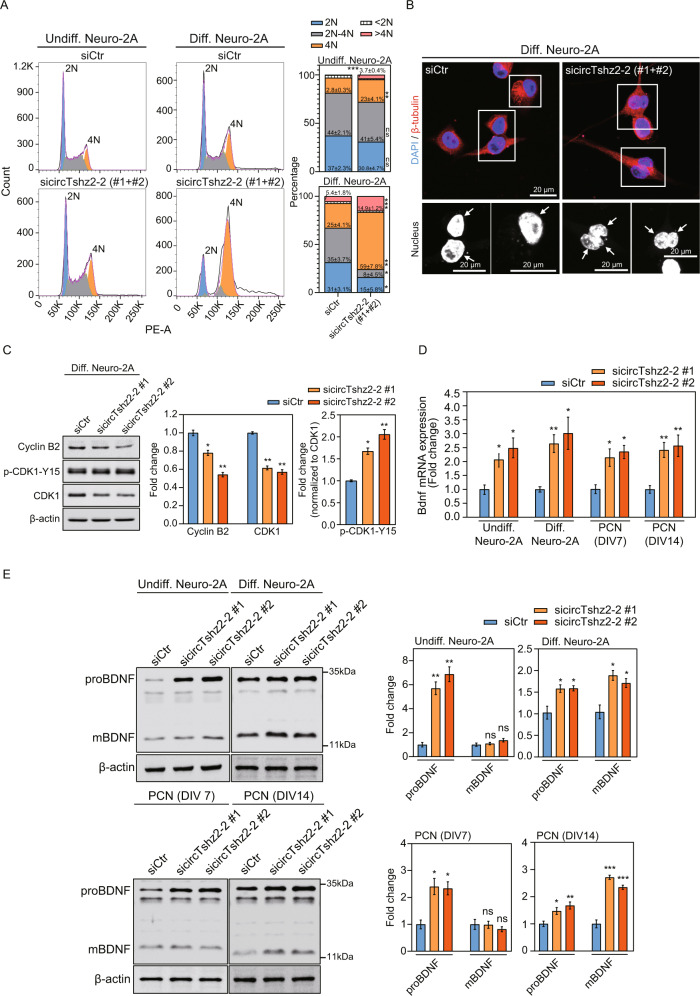
Cell cycle regulation by circTshz2-2. **A** The distribution of cell cycle phase following circTshz2-2 knockdown in undifferentiated (Undiff.) Neuro-2A cells at day 3 and differentiated (Diff.) Neuro-2A cells at day 5. The graph in the left panel depicts the representative results from three experiments (*n* = 3), and the data were averaged to produce the histogram in the right panel (mean ± SEM). Percentages of cells in each phase were calculated using FlowJo software and siCtr indicates the negative control siRNA. PE-A indicates phycoerythrin-area, while 2N, 2N–4N, 4N, <2N, and >4N denote G1, S, G2/M, sub-G1, and any phase greater than G2/M, respectively. **B** The changes in nuclear morphology and neurite length following circTshz2-2 knockdown in differentiated Neuro-2A cells at day 5. These data are depicted using representative cells from three independent cultures (*n* = 3) and the beta-tubulin and nuclei (arrow) are indicated by red and blue (DAPI), respectively. The rectangular box is a magnified field of view. In **A** and **B**, the data from the sicircTshz2-2 cells represent the outcomes of mixing two different siRNAs against circTshz2-2 (#1 and #2). **C** The changes in the protein levels of Cyclin B2, CDK1, and p-CDK1-Y15 following circTshz2-2 knockdown in differentiated Neuro-2A cells at day 5. These expression changes are depicted as the mean ± SEM (*n* = 3). p-CDK1-Y15 indicates the CDK1 protein phosphorylated at tyrosine 15. The level of p-CDK1-Y15 was normalized against CDK1. **D** The changes in *Bdnf* transcription following circTshz2-2 knockdown in neuronal cells were measured and are described as the mean ± SEM (*n* = 3). PCN indicates mouse primary cortical neurons. DIV indicates the days in vitro. **E** The changes in proBDNF and mBDNF protein levels following circTshz2-2 knockdown in neuronal cells were evaluated and are depicted as the mean ± SEM (*n* = 3). In **A**, and **C**–**E**, statistical significance was analyzed using an unpaired two-tailed *t-*test with Welch’s correction; ns not significant, *p < 0.05, **p < 0.01, ***p < 0.001.

A previous study reported that neuronal cells arrested at G0/G1 or G2/M phases become long neurite-bearing neurons during differentiation and development [29]. These findings indicate that the neurons, which are prototypical postmitotic cells, can maintain and regulate neurite elongation and complexity via their regulation of the cell cycle. We first compared the cell cycle distribution between undifferentiated and differentiated Neuro-2A cells using propidium iodide staining and flow cytometry. We noted an increase in the percentage of differentiated cells in both the G2/M (4N) and polyploidy phases (>4N) and a reduction in the proportion of cells in the G1 (2N), S (2N–4N), and Sub-G1 (<2N) phases when compared to undifferentiated cells (Fig. 3A). To examine the role of circTshz2-2 during cell cycle regulation, circTshz2-2 expression was suppressed using siRNAs in both undifferentiated and differentiated Neuro-2A cells, respectively. Inhibition of circTshz2-2 significantly increased the proportion of cells in the G2/M (4N) and polyploidy phases (>4N) while reducing the number of cells in the G1 (2N), S (2N–4N), and sub-G1 (<2N) phases compared to the negative control in both the undifferentiated and differentiated cells. This indicates that circTshz2-2 knockdown arrests the cell cycle at the G2/M phase and causes neurons to remain in the polyploid states.

This polyploidy is categorized as nuclear polyploidy (mononucleated) or cellular polyploidy (binucleated and more) depending on endoreplication, mitotic slippage, and cytokinesis failure in the absence of mitosis [30]. To characterize which polyploidy occurs following the circTshz2-2 knockdown, we stained the DNA using 4′,6-diamidino-2-phenylindole (DAPI), and the microtubules using a beta-tubulin antibody in circTshz2-2-depleted undifferentiated and differentiated Neuro-2A cells, respectively. In undifferentiated conditions, most cells had a U-shaped, which is defined as putative metaphase [31], and mononucleated nucleus by circTshz2-2 knockdown, although a small number of cells had a binucleated nucleus (Supplementary Fig. S12). However, we observed mostly binucleated and trinucleated cells bearing long neurites after inhibition of circTshz2-2 in differentiated cells (Fig. 3B), indicating that this circRNA has some role in regulating polyploidy following G2/M cycle arrest in differentiated cells. We also tested the changes in nuclear morphology after the circTshz2-2 overexpression. However, the nuclear morphology was indistinguishable between the cells transfected with the control and overexpression vector. It is plausible that the defect in nuclear morphology is observed only when the amount of circTshz2-2 is below a certain amount. Otherwise, the circTshz2-2 overexpressed cells may not be in the G2/M phase, considering the increased expression of genes associated with cell division and chromosome segregation in these cells (Supplementary Fig. S11).

Mitotic failure occurs when the kinetochore, a spindle-binding complex to segregate chromosome, is not properly assembled, and consequently, endoreplication, mitotic slippage, and cytokinesis failure occur [32]. The inhibition of circTshz2-2 markedly reduced the expression of genes related to kinetochore assembly (Ndc80, Spc24) based on our RNA-seq dataset (Fig. 2C), suggesting that the suppression of circTshz2-2 arrests the cell cycle at the G2/M phase and increases the proportion of cells presenting with polyploidy due to kinetochore malfunction.

### CircTshz2-2 regulates Cyclin B2/CDK1 and BDNF expression

Both Cyclin B proteins and CDK1 are specific to the G2/M checkpoint during cell cycle regulation. Downregulation in the expression or activity of Cyclin B and CDK1 results in cell cycle arrest at G2/M [29]. Our RNA-seq and qRT-PCR data from circTshz2-2-depleted cells showed significant decreases in the genes related to the regulation of the G2/M phase checkpoint (*Ccnb2* and *Cdk1*), suggesting that the G2/M phase arrest induced by circTshz2-2 knockdown might be related to changes in Cyclin B2 and CDK1 expression. To confirm this, differentiated Neuro-2A cells were transfected with circTshz2-2 siRNAs. CircTshz2-2 knockdown reduced the expression of Cyclin B2 and CDK1 in differentiated cells (Fig. 3C). A previous study found that phosphorylation of CDK1 at tyrosine 15 also inactivates the Cyclin B/CDK1 complex resulting in G2/M phase arrest in tetraploid neurons [33]. Our results also showed that circTshz2-2 knockdown increased the phosphorylation status of CDK1 at tyrosine 15 in differentiated tetraploid Neuro-2A cells (Fig. 3C). We again verified the Cyclin B2 and CDK1 activity in differentiated cells after circTshz2-2 overexpression. The expression of Cyclin B2 and CDK1 was significantly increased following circTshz2-2 overexpression in differentiated cells. However, there was no difference in the phosphorylation of CDK1 at the tyrosine 15 site between the cells transfected with the control and overexpression vector (Supplementary Fig. S13A). These results demonstrate that circTshz2-2 regulates cell cycle arrest at the G2/M phase by regulating the amount of G2/M checkpoint proteins including Cyclin B2 and CDK1.

A recent report suggested that brain-derived neurotrophic factor (BDNF) arrests the cell cycle at the G2/M phase through its regulation of Cyclin B/CDK1 activity in differentiating tetraploid retinal ganglion cells during normal development [33]. To examine whether the G2/M arrest associated with circTshz2-2 knockdown depends on BDNF expression in the neuron, Neuro-2A cells and mouse primary cortical neurons were transfected with the circTshz2-2 siRNAs and evaluated with qRT-PCR. CircTshz2-2 knockdown significantly increased Bdnf mRNA expression in undifferentiated and differentiated Neuro-2A cells and primary cortical neurons at DIV 7 (7 days in vitro) and DIV 14 (Fig. 3D), suggesting that circTshz2-2 knockdown increases *Bdnf* transcription in the neuron. The Bdnf mRNA is translated into precursor BDNF (proBDNF) protein and is proteolytically processed to produce the mature form of BDNF (mBDNF) by various proteases [34]. To examine which form of BDNF protein is modulated by circTshz2-2 knockdown, the Neuro-2A cells and mouse primary cortical neurons were transfected with the circTshz2-2 siRNAs and subjected to western blot. CircTshz2-2 knockdown increased proBDNF levels in undifferentiated Neuro-2A and primary cortical neurons at DIV 7 (Fig. 3E). However, it did not change the mBDNF level in either cell type, indicating that circTshz2-2 knockdown only increases proBDNF expression in undifferentiated and immature neurons. On the contrary, circTshz2-2 knockdown markedly increased both proBDNF and mBDNF expression in differentiated Neuro-2A and primary cortical neurons at DIV 14 (Fig. 3E), indicating that circTshz2-2 regulates both proBDNF and mBDNF levels in the differentiated and mature neuron. To confirm whether proBDNF and mBDNF levels are affected by circTshz2-2 overexpression, differentiated Neuro-2A cells were transfected with circTshz2-2 overexpression vectors, and then western blot was performed. CircTshz2-2 overexpression significantly decreased both proBDNF and mBDNF levels in differentiated cells (Supplementary Fig. S13B), confirming the regulation of BDNF expression by circTshz2-2. Our results suggest that circTshz2-2 regulates cell cycle arrest at the G2/M phase by regulating Cyclin B2/CDK1 and BDNF expression.

### CircTshz2-2 regulates BDNF expression via its modulation of the YY1 transcriptional repressor complex

**Fig. 4:**
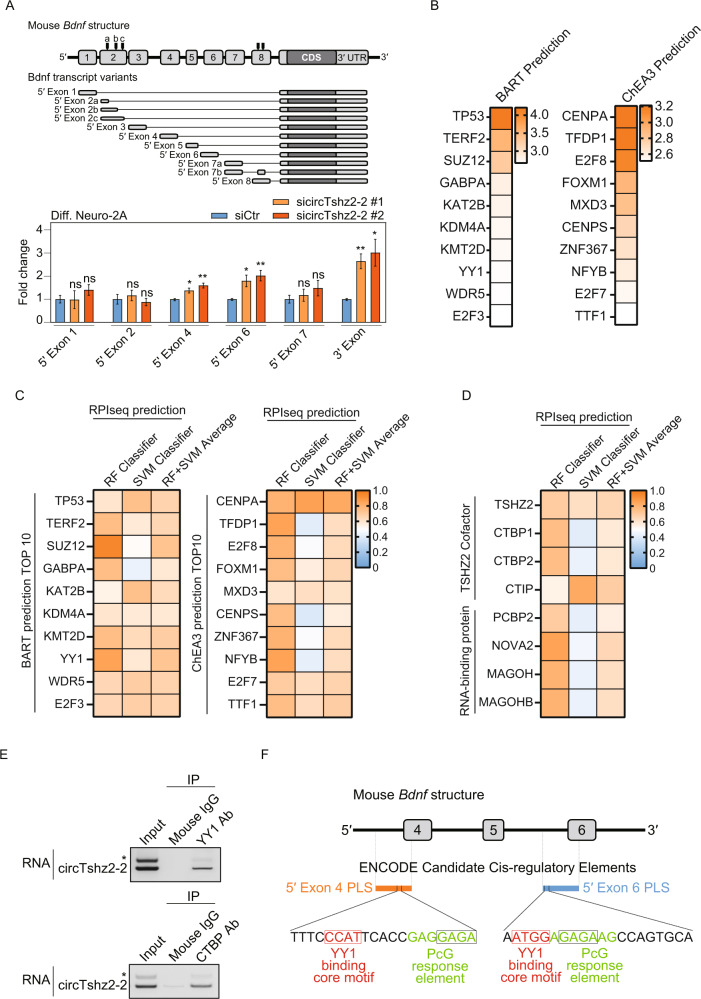
Regulation of *Bdnf* transcription by circTshz2-2 and the YY1 transcriptional repressor complex. **A** Illustration describing the mouse *Bdnf* gene and transcript variants, and the changes in the levels of Bdnf mRNA isoforms following circTshz2-2 knockdown in differentiated (Diff.) Neuro-2A cells at day 5. The alternative splicing sites in the mouse *Bdnf* gene are shown by the black bar. The data are reported as the mean ± SEM (*n* = 3). siCtr indicates the negative control siRNA and statistical significance was analyzed using an unpaired two-tailed *t-*test with Welch’s correction; ns not significant, *p < 0.05, **p < 0.01, ***p < 0.001. **B** The prediction of possible protein factors which may regulate the transcription of those genes affected by circTshz2-2-depletion in the RNA-seq data. The color bar represents the Irwin-Hall *p* value for the BART evaluation and the Integrated Score Rank from ChEA3, respectively. **C** The interaction probability between circTshz2-2 and specific transcriptional regulators was predicted using RPIseq. The color bar represents the interaction probability score (0–1). RF and SVM indicate random forest and support vector forest, respectively. **D** The interaction probability between circTshz2-2 and TSHZ2 co-factors or RNA-binding proteins was predicted by RPIseq. The color bar indicates the interaction probability score (0–1). **E** Confirmation of the RNA-protein interactions between circTshz2-2 and YY1 (upper) or CTBP (lower) in differentiated Neuro-2A cells at day 5. The data are from a representative experiment (*n* = 3). The band marked with an asterisk is circTshz2-1. IP indicates immunoprecipitation. **F** Illustration of the promoter-like signature (PLS) in the sequences of the mouse *Bdnf* gene from exons 4 through 6. The PLS data were obtained from the Candidate Cis-regulatory Elements database in ENCODE. The YY1 binding core motif and polycomb group (PcG) response elements are highlighted in red and green, respectively.

*Bdnf* is transcribed as diverse isoforms by various transcriptional regulators [35]. To determine the working mechanism underlying circTshz2-2 mediated regulation of BDNF expression, we first evaluated which Bdnf mRNA isoforms increased following the circTshz2-2 knockdown. Among the 11 Bdnf isoforms present in the mouse (Fig. 4A and Supplementary Fig. S14A), only six isoforms were expressed in differentiated Neuro-2A cells, with these isoforms beginning at the 5′ end of exons 1, 2, 4, 6, and 7, respectively (Supplementary Fig. S14B). The inhibition of circTshz2-2 expression increased the expression of the isoforms starting in exons 4 and 6 (Fig. 4A), indicating that circTshz2-2 knockdown increases Bdnf mRNA expression by modulating the transcription of these two *Bdnf* isoforms. Again, we observed that these two Bdnf isoforms significantly decreased in differentiated cells transfected with circTshz2-2 overexpression vector compared to control vector-treated cells (Supplementary Fig. S14C), confirming that circTshz2-2 regulates the Bdnf transcription by modulating these two isoforms in the neuron.

To elucidate the mechanism underlying this transcriptional regulation, we first went on to identify any transcription factor or chromatin regulator which commonly regulates the differentially expressed genes identified in our RNA-seq data. Using two publicly available prediction tools, BART and ChEA3, we first investigated the transcription factors and chromatin regulators involved in controlling the transcription of all 143 genes related to the cell cycle in our RNA-seq data (Fig. 2A). The top 10 transcription factors based on the prediction scores from both BART and ChEA3 were then selected for further evaluation (Fig. 4B). We then used RNA-protein interaction prediction tool RPIseq to identify those factors most likely to interact with circTshz2-2 (Fig. 4C). We also included any RNA-binding proteins experiencing differential expression following the circTshz2-2 knockdown, and any of the co-factors related to *Tshz2* in these evaluations (Fig. 4D). The factors with the highest binding probability, more than 0.5, in both classifiers were then selected from the RPIseq results (Fig. 4C, D). Among the various factors identified, polycomb protein SUZ12, C-terminal binding protein (CTBP), and Ying Yang 1 (YY1) have all been previously identified as regulators of BDNF. These proteins belong to the polycomb repressive complex [36], suggesting that they act as transcriptional repressors of BDNF. The detailed mechanism of CTBP in regulating *BDNF* transcription has not been reported, however, it is known that its regulation of the human *BDNF* transcripts is facilitated by its binding of a DNA region between exons 4 and 6, as shown in the human ChIP-seq dataset (Supplementary Fig. S15) [37]. Importantly, it was reported that YY1 conditional knockout increases *Bdnf* expression and causes cytokinesis failure, resulting in binucleated tetraploids during embryonic development [38]. Thus, these previous results reported a phenotype similar to those described in our results where circTshz2-2 knockdown increases *Bdnf* transcription, and tetraploidy and polyploidy by kinetochore malfunction in the neuron (Fig. 3).

Given this, we moved on to complete a series of RNA-IP assays to verify whether circTshz2-2 binds SUZ12, CTBP, and YY1. Interestingly, circTshz2-2 bound with YY1 and CTBP but not with SUZ12 (Fig. 4E and Supplementary Fig. S16A, B), suggesting that circTshz2-2 acts as a co-factor for YY1 and CTBP in regulating *Bdnf* transcription. It has been reported that YY1 is known to recruit a CTBP co-repressor to act as its partner in transcriptional repressor complexes, and CTBP has a binding partner, CTBP-interacting protein (CTIP) [36, 39]. We observed that circTshz2-2 was also co-precipitated in a complex which contains CTIP in differentiated cells (Supplementary Fig. S16C), indicating that circTshz2-2 likely acts as a repressive co-factor in the YY1-CTBP-CTIP polycomb transcriptional repressive complex. In addition, it was recently reported that YY1 binds to a core motif sequence containing CCAT and ATGG, and that the polycomb group complex binds to a GAGA consensus sequence, resulting in gene repression [40–42]. It has also been reported that the polycomb repressor complex binds to the *Bdnf* promoter near exon 6 to suppress *Bdnf* transcription [43]. The data from the ENCODE Cis-Regulatory data Elements (cCREs) database also shows that the YY1 transcriptional repressive complex binds to the *Bdnf* promoter region of exons 4 and 6 (Fig. 4F) [44]. Therefore, our results indicate that the YY1-CTBP-CTIP polycomb repressive complex acts in concert with circTshz2-2 to bind to the *Bdnf* promoter and suppress *Bdnf* transcription.

### CircTshz2-2 regulates G2/M cell cycle arrest through BDNF/TrkB signaling pathway

**Fig. 5:**
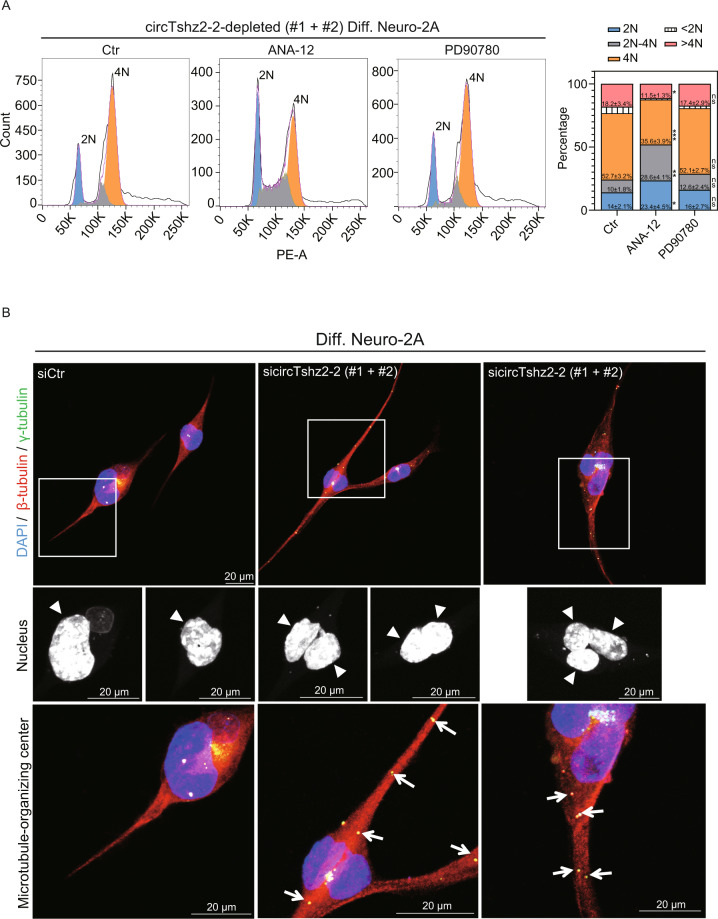
Regulation of the cell cycle and the re-location of the microtubule-organizing center by the circTshz2-2/TrkB signaling pathway. **A** Changes in the cell cycle in circTshz2-2-depleted differentiated Neuro-2A cells at day 5 following treatment with a TrkB antagonist (ANA-12) and p75NTR inhibitor (PD90780). The data represent three independent repeats (*n* = 3) and are expressed as the mean ± SEM. Percentages of cells in each phase were calculated using FlowJo software. siCtr indicates the negative control siRNA. PE-A indicates phycoerythrin-area. 2N, 2N–4N, 4N, <2N, and >4N indicate the phase of G1, S, G2/M, sub-G1, and phases greater than G2/M, respectively. Statistical significance was analyzed using an unpaired two-tailed *t-*test with Welch’s correction; ns not significant, *p < 0.05, **p < 0.01, ***p < 0.001. **B** The changes in the nuclear morphology and the re-location of the microtubule-organizing center following circTshz2-2 knockdown in differentiated Neuro-2A cells at day 5. The beta-tubulin, gamma-tubulin (triangle), and nuclei (arrow) are indicated by red, green, and blue, respectively. The data are shown as representative cells from three independent cultures (*n* = 3). In **A** and **B**, the sicircTshz2-2 data are derived from cells treated with a mix of two different siRNAs against circTshz2-2 (#1 and #2).

It has been reported that proBDNF and mBDNF stimulate their downstream signaling pathway via their binding to their receptors, p75 neurotrophin receptor (p75NTR) and tyrosine receptor kinase B (TrkB), respectively [45]. We examined whether the G2/M arrest facilitated by circTshz2-2 knockdown depends on the activation of the BDNF receptors in differentiated Neuro-2A cells. The cells were transfected with circTshz2-2 siRNAs and then treated with a TrkB antagonist (ANA-12) and a p75NTR inhibitor (PD90780), respectively. These cells were then subjected to another analysis of their cell cycle distribution. TrkB inhibition increased the proportion of cells in the G1 (2N) and S (2N–4N) phases and decreased the proportion of cells in the G2/M (4N) and polyploidy (>4N) phases in circTshz2-2-depleted cells compared to the control (Fig. 5A). However, p75NTR inhibition did not affect the cell cycle distribution, indicating that the cell cycle arrest at the G2/M phase facilitated by circTshz2-2 knockdown depends on the TrkB signaling pathway but not the p75NTR signaling pathway. Given this, these results indicate that circTshz2-2 regulates cell cycle arrest at the G2/M phase via the mBDNF/TrkB signaling pathway.

### Downregulation of circTshz2-2 re-locates the microtubule-organizing center to the neurite

The microtubule-organizing center (MTOC) is a major site of microtubule nucleation and is primarily responsible for creating the microtubule spindle needed to divide chromosomes during the cytokinesis process [46]. However, during neurite elongation, the MTOC also serves to generate and rearrange the neurite microtubules [47]. We observed that the inhibition of circTshz2-2 increased neurite length, induced G2/M cell cycle arrest, and produced an increase in binucleated and trinucleated polyploidy by promoting kinetochore malfunction in the neuron (Figs. 1 and 3). Therefore, we checked whether the MTOC, which was not used during cell division, is re-located to generate neurite microtubules in circTshz2-2-depleted cells. The cells were transfected with circTshz2-2 siRNAs and stained with DAPI to identify the nucleus, an anti-gamma-tubulin antibody to identify the MTOC, and an anti-beta-tubulin antibody for the microtubules, respectively. Interestingly, the MTOCs were evenly distributed within the relatively long neurite in circTshz2-2-depleted neurons exhibiting binucleated and trinucleated polyploidy (Fig. 5B). The control cells also demonstrated the production of new neurites, but the distribution of the MTOC within the neurite was not observed. Therefore, these results indicate that circTshz2-2 regulates neurite elongation through the re-location of the MTOC involved in the G2/M phase.

### CircTshz2-2 regulates spatial memory and cell cycle proteins in wild-type and obese mice

**Fig. 6:**
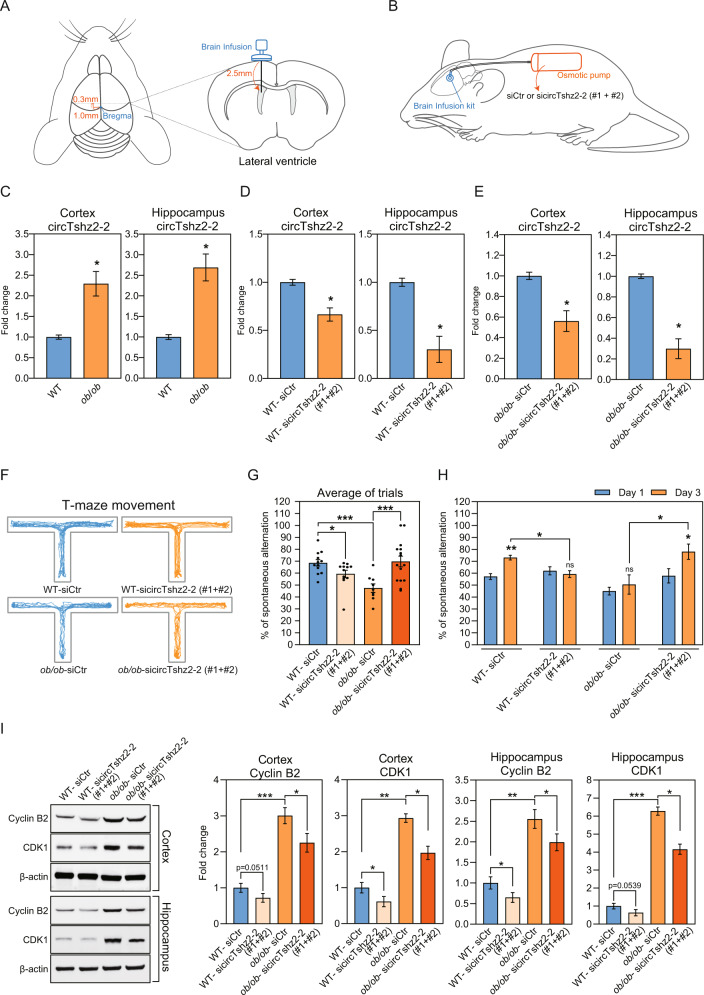
Regulation of spatial memory by circTshz2-2 in wild-type and obese mice. **A** Illustration displaying the infusion location of brain infusion kit and osmotic pump on the brain. The infused position at the lateral ventricle is mediolateral 1.0 mm from bregma, anteroposterior 0.3 mm, and dorsoventral 2.5 mm, in the order. **B** Illustration displaying the implantation of osmotic pump containing a solution (control siRNA or circTshz2-2 siRNAs). The brain infusion kit was inserted into the lateral ventricle. The osmotic pump was fixed in the dorsal subcutaneous area. sicircTshz2-2 (#1 + #2) indicates a mixture of two independent circTshz2-2 siRNAs. **C** The expression of circTshz2-2 in the brain cortex and hippocampus of wild-type (WT) and transgenic obese (*ob*/*ob*) mice, respectively, is described as the mean ± SEM (*n* = 5). **D** Changes in the expression of circTshz2-2 following circTshz2-2 depletion in the brain cortex and hippocampus of wild-type mice are described as the mean ± SEM (*n* = 5). **E** Changes in the expression of circTshz2-2 following circTshz2-2 depletion in the brain cortex and hippocampus of obese mice are described as the mean ± SEM (*n* = 5). **F** Illustration describing the movement of wild-type and obese mice in the T-maze apparatus after infusion of either control siRNA or circTshz2-2 siRNA mixtures. The representative trace indicates the movement for 5 min of five mice per group. **G** Changes in the percentage of spontaneous alternation following circTshz2-2 depletion in the wild-type and obese mice are described as the mean ± SEM (*n* = 5). It displays the average percentage of spontaneous alternation obtained from days 1 and 3 among the trials. The dots on the bar mean a percentage of spontaneous alternation of the mice in each group. **H** Changes in the percentage of spontaneous alternation at days 1 and 3 following circTshz2-2 depletion in wild-type and obese mice are described as the mean ± SEM (*n* = 5). **I** Changes in the level of Cyclin B2 and CDK1 following circTshz2-2 depletion in the brain cortex and hippocampus of wild-type and obese mice are represented as the mean ± SEM (*n* = 5). In **D**–**I**, WT-siCtr indicates the control siRNA-infused wild-type mice. WT-sicircTshz2-2 (#1 + #2) indicates the wild-type mice infused with the mixture of two independent circTshz2-2 siRNAs. *ob*/*ob*-siCtr indicates control siRNA-infused obese mice. *ob*/*ob*-sicircTshz2-2 (#1 + #2) indicates the obese mice infused with the mixture of two independent circTshz2-2 siRNAs. In **C**–**E**, and **G**–**I**, statistical significance was determined using an unpaired two-tailed *t*-test with Welch’s correction; ns not significant, *p < 0.05, **p < 0.01, ***p < 0.001.

The changes in the neuronal structure and cell cycle are linked to neurodegeneration and memory function in the brain [48–50]. Therefore, we evaluated the memory function of wild-type and obese (*ob*/*ob*) mice after the knockdown of circTshz2-2 in the brain. To suppress circTshz2-2 expression in the brain of mice, two circTshz2-2 siRNA mixtures were infused into a lateral ventricle of the mouse brain (Fig. 6A, B). First, we observed a higher expression of circTshz2-2 in the cortex and hippocampus of obese mice than that of wild-type mice (Fig. 6C). After infusion of circTshz2-2 siRNAs, the circTshz2-2 expression was efficiently reduced in the cortex and hippocampus of both wild-type and obese mice (Fig. 6D, E). To evaluate whether circTshz2-2 dysregulation affected the memory function, we checked the spatial memory of these mice through a T-maze test. The T-maze is used for spontaneous alternation tests based on the willingness of rodents to explore a new environment, reflecting the spatial memory about the previously visited environment [51]. The movement and locomotor activities (the number of entrances to arm, total distance, velocity, and immobility time) of each subject in T-maze were recorded to evaluate spatial memory (Fig. 6F and Supplementary Fig. S17). Although we found a significant reduction in locomotor activities in the obese mice compared to the wild-type mice (Supplementary Fig. S17A–D), there was no difference in locomotor activities between the mice infused with control and circTshz2-2 siRNAs, regardless of either wild-type or obese mice (Supplementary Fig. S17E–H), indicating that surgical procedure and circTshz2-2 depletion did not affect the movement and locomotor activities in each of wild-type and obese mice. We observed impairment of spatial memory in obese mice compared to wild-type mice (Fig. 6G), indicating the obesity-induced spatial memory impairment. Interestingly, circTshz2-2 depletion impaired the spatial memory in wild-type mice but improved it in obese mice (Fig. 6G). When the spatial memory capacity was compared between days 1 and 3, it was improved on day 3 compared to day 1 in the wild-type mice infused with control siRNA. However, it did not improve on day 3 compared to day 1 in the wild-type mice infused with circTshz2-2 siRNAs (Fig. 6H), suggesting that circTshz2-2 depletion interfered with the spatial learning during trials in wild-type mice. For obese mice, the spatial memory did not improve on day 3 compared to day 1 in control siRNA-infused mice. However, it was noticeably enhanced on day 3 compared to day 1 in circTshz2-2 siRNAs-infused mice (Fig. 6H), indicating that circTshz2-2 depletion improves spatial learning during trials in obese mice. Collectively, our data showed that although the circTshz2-2 dysregulation impaired the spatial memory in normal conditions, the suppression of circTshz2-2 improved spatial memory in the brain of obese mice where this circRNA is upregulated (Fig. 6C).

A previous result showed that Cyclin B2 and CDK1 increased in the condition of neurodegeneration and memory impairment [52]. However, these proteins have not been measured in obesity-induced memory impairment. Therefore, we examined the expression of Cyclin B2 and CDK1 in the cortex and hippocampus of wild-type and obese mice after circTshz2-2 depletion. First, we observed higher Cyclin B2 and CDK1 expression in the cortex and hippocampus of obese mice than wild-type mice (Fig. 6I), indicating that G2/M checkpoint proteins increase under the condition of obesity-induced neurodegeneration. Importantly, Cyclin B2 and CDK1 expression was significantly decreased following circTshz2-2 suppression in the cortex and hippocampus of both wild-type and obese mice (Fig. 6I). These results imply that circTshz2-2 regulates spatial memory by modulating the amount of G2/M checkpoint proteins. In addition, circTshz2-2 depletion compromised the increase of these proteins in obesity conditions. Taken together, our data propose the critical potential of circTshz2-2 on memory improvement in the obese brain.

## Discussion

Here, we investigate the functions of circTshz2-2 on the regulation of neuronal function in the obese brain. Given our data, neurons showed high levels of circTshz2-2 expression, and the inhibition of circTshz2-2 increased neurite length and neural complexity, indicating that circTshz2-2 plays a repressive role in the development of the neural structure. CircTshz2-2 suppresses *Bdnf* transcription by binding to the YY1 transcriptional repressive complex, whereas circTshz2-2 dysregulation increases BDNF expression by preventing the formation of the YY1 transcriptional repressor complex. Increased BDNF expression induces G2/M cell cycle arrest via the TrkB/Cyclin B2/CDK1 signaling pathway, and circTshz2-2 dysregulation induced kinetochore malfunction producing polyploid neurons. This dysregulation also resulted in neurite elongation via the relocation of the cytokinesis-related MTOC within the neurite (Supplementary Fig. S18), indicating that circTshz2-2 affects neuronal structure through regulation of the G2/M cell cycle during the neuronal development process. Furthermore, circTshz2-2 contributes to spatial memory capacity by modulating the expression of G2/M checkpoint protein in the brain. The expression of Cyclin B2 and CDK1 was suppressed by circTshz2-2 depletion, which regulated spatial memory capacity in the normal and obese brain. Our data revealed the roles of an obesity-related circRNA, circTshz2-2, in regulating neuronal cell cycle and spatial memory capacity in the brain.

We proceeded with transcriptome analysis to identify the mechanism underlying the increases in neurite elongation and complexity following circTshz2-2 dysregulation. The decreased genes were primarily associated with cell cycle regulation, especially those associated with cell division. We did not experimentally validate the genes related to neuronal function because the main function of the YY1 transcriptional repressor complex and its interactions with circTshz2-2 were shown to be related to the cell cycle and division process via their regulation of BDNF expression. Interestingly, many studies have reported that the genes associated with neuronal function changed by circTshz2-2 knockdown play an important role in metabolic syndromes such as obesity and diabetes mellitus [53]. The increased serum concentration of beta-2 microglobulin (B2M) is used as a clinical marker for diabetes mellitus [54], and high-mobility group protein B2 (HMGB2) is used as a diagnostic marker for both obese and diabetic patients [55]. Matrix metalloproteinase-19 has been found to be significantly upregulated in the adipose tissues of obese and diabetic mice [56]. Protein tyrosine phosphatase receptor type T is known to prevent obesity by regulating the metabolism in high-fat-diet mice [57], and metabolic imbalances reduce the expression of proto-oncogene protein WNT3 in obese and diabetic samples [58]. Given the fact that circTshz2-2 is an obesity-related circRNA, it is interesting that circTshz2-2 dysregulation alters the expression of genes related to both systemic metabolic syndromes and neuronal function related to neuropathology in the brain.

Our results indicate that the inhibition of circTshz2-2 increases *Bdnf* transcription and proBDNF level in immature neurons. ProBDNF primarily activates the p75NTR signaling pathway [59]. Thus, we expected that G2/M cell cycle arrest following circTshz2-2 dysregulation would be linked to the proBDNF/p75NTR signaling pathway. However, we observed that p75NTR signaling did not control G2/M cell cycle arrest in the neuron. We hypothesize that this result is explained by the cell surface expression of p75NTR. It has been reported that p75NTR is mainly expressed on the surface of cells in the G1, S, and G2 phases [60]. The observation that most circTshz2-2 knockdown cells remain in the G2/M phase, especially the mitotic phase, suggests that p75NTR may not be available on the cell surface meaning that the increased proBDNF levels cannot affect p75NTR signaling in these cells.

Furthermore, inhibition of circTshz2-2 increased mBDNF levels in differentiated and mature neurons, suggesting that circTshz2-2 itself is not involved in proBDNF processing. In general, serine protease Furin, prohormone convertases, and matrix metalloproteases (MMPs) are involved in the proteolytic cleavage of proBDNF into mBDNF [59, 61, 62]. The altered mBDNF level is thought to be related to the increased availability of the proBDNF precursor during the differentiation and maturation of the neuron. It has been reported that the expression and secretion of various MMPs, MMP-2 and MMP-9, are increased by retinoic acid [63, 64]. Several studies have also reported that mBDNF is proteolytically processed during the maturation of primary neurons to promote the activity-dependent maintenance of dendritic spine morphogenesis [65, 66]. Therefore, the increased proBDNF levels following circTshz2-2 dysregulation might increase mBDNF levels in differentiated and mature neurons, thereby controlling the cell cycle through the TrkB receptor.

CircTshz2-2 expression increased in differentiated and mature neurons, and ultimately circTshz2-2 plays an important role in neurite elongation and complexity during differentiation and maturation. Considering that circTshz2-2 suppression increased neurite length and complexity, this circRNA is expected to act as a repressor for neurite elongation and complexity. Maintaining neurite growth and complexity is important for normal brain development and function [67]. If the circTshz2-2 expression is markedly changed in the neuron, it is thought that it could affect neurological diseases by inducing abnormal neurite outgrowth and branching. Abnormal neurite outgrowth is critical for neurodegenerative and neurodevelopmental diseases. It was reported that the trans-differentiation of fibroblasts obtained from patients with Huntington’s disease into neuron-like cells shows abnormal neurite branching compared to those from normal individuals [68]. It has also been reported that when amyloid-beta, which is a hallmark of Alzheimer’s disease, is overexpressed in the primary neurons of *Drosophila melanogaster*, these cells exhibit abnormal neurite outgrowth and branching [69]. The dysregulation of neurodevelopmental genes was also observed in patients with autism showing abnormal neurite outgrowth [70]. Interestingly, it has also been reported that maternal obesity and diabetes led to fetal autism with abnormal neurite outgrowth, resulting in neuronal network hyperexcitability [71], suggesting that there might be a role for obesity-related circRNAs, like circTshz2-2, in the pathophysiology of neurological diseases. Taken together, we propose that circTshz2-2 dysregulation affects neural morphology and structure, leading to the onset of neurological diseases.

Like the highly differentiated tissues such as heart muscle and placenta [72], terminally differentiated neurons also exhibit polyploidy in some brain regions such as the cortex where neurons need to communicate very far away [73]. These polyploid neurons are relatively large and have long neurites in the brain, and it is protected from cell death after brain damage [74]. Although there is no report about the relationship of polyploidy frequency between the healthy brain and obese brain, it was reported that obesity leads to neuronal damage through abnormal CDKs-mediated cell cycle re-entry in the mediobasal hypothalamus [75]. In addition, another study showed that downregulation of the expression of Cyclin B and CDK1 results in cell cycle arrest at the G2/M phase [29]. Therefore, we assume that abnormal expression of Cyclin/CDKs results in the low frequency of polyploidy in the brain with obesity because circTshz2-2 suppression reduced the expression of Cyclin B2 and CDK1 in the brain cortex and hippocampus of obese mice.

It was reported that some neurons undergo self-fusion during neural development and brain repair [76], under certain stress conditions such as stroke [77], and by experimental artifact following siRNA transfection [78]. Accordingly, an alternative explanation could be raised that the neuronal polyploidy that we observed was made by cell–cell fusion under a stress condition other than the direct effect from circTshz2-2 depletion. However, because we confirmed that circTshz2-2 regulated diverse genes related to cell division, chromosome segregation, and kinetochore assembly, and influenced cell cycle distribution, it is certain that circTshz2-2 is an important regulator on the G2/M cycle, which is directly linked to polyploidy formation in the neuron.

For decades, many studies have shown that metabolic syndromes such as obesity can be a fundamental cause of the development of neurodegenerative disease [79]. However, no study has reported the dysregulation of the neuronal cell cycle in the brain with obesity. Abnormal regulation of the cell cycle was observed in the brain by regulating the neuronal structure, synapse formation, and memory function [80, 81]. Other studies reported the cell cycle re-entry and dysregulation in neurodegenerative diseases such as mild cognitive impairment and Alzheimer’s disease [50, 82]. In this study, we observed that circTshz2-2 is highly expressed in obese mice brains and regulates the cell cycle in neurons. Based on the results that circTshz2-2 depletion improved spatial memory capacity by regulating cell cycle proteins in the obese brain, we expect the potential to target this circRNA for the improvement of cognitive decline and memory impairment in the patient with obesity.

## Supplementary information

Supplementary figures

Supplementary materials and methods

Supplementary Table S1

Supplementary Table S2

Supplementary Table S3
