# Supplementary material for: Obesity-linked circular RNA circTshz2-2 regulates the neuronal cell cycle and spatial memory in the brain
Source: Mol Psychiatry. 2021 Sep 24;26(11):6350–64. doi: 10.1038/s41380-021-01303-x (PMC8760052; doi:10.1038/s41380-021-01303-x)
Supplement: Supplementary file 1 — Supplementary figures [file 41380_2021_1303_MOESM1_ESM.docx]

Supplementary Figures for

**Obesity-linked circular RNA circTshz2-2 regulates the neuronal cell cycle and spatial memory in the brain**

Gwangho Yoon, Yeong-Hwan Lim, Danbi Jo, Juhee Ryu, Juhyun Song*, Young-Kook Kim*

*Corresponding author. Email: Young-Kook Kim: ykk@jnu.ac.kr; Juhyun Song: Juhyunsong@chonnam.ac.kr

**This file includes:**

Supplementary Fig. S1 to S18

Supplementary Tables S1 to S3 (Data are available online)

**Supplementary Fig. S1**


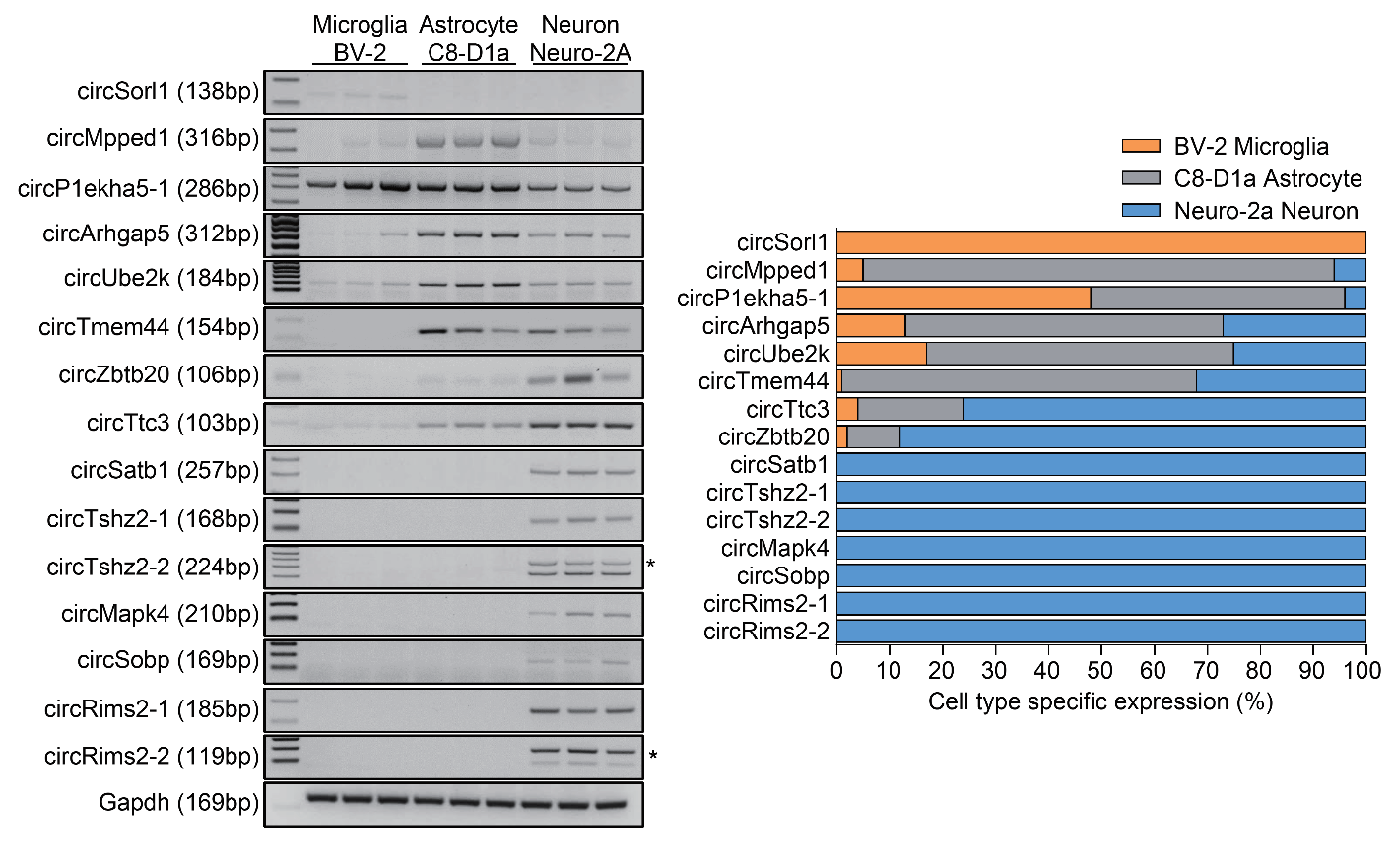


**Supplementary Fig. S2**


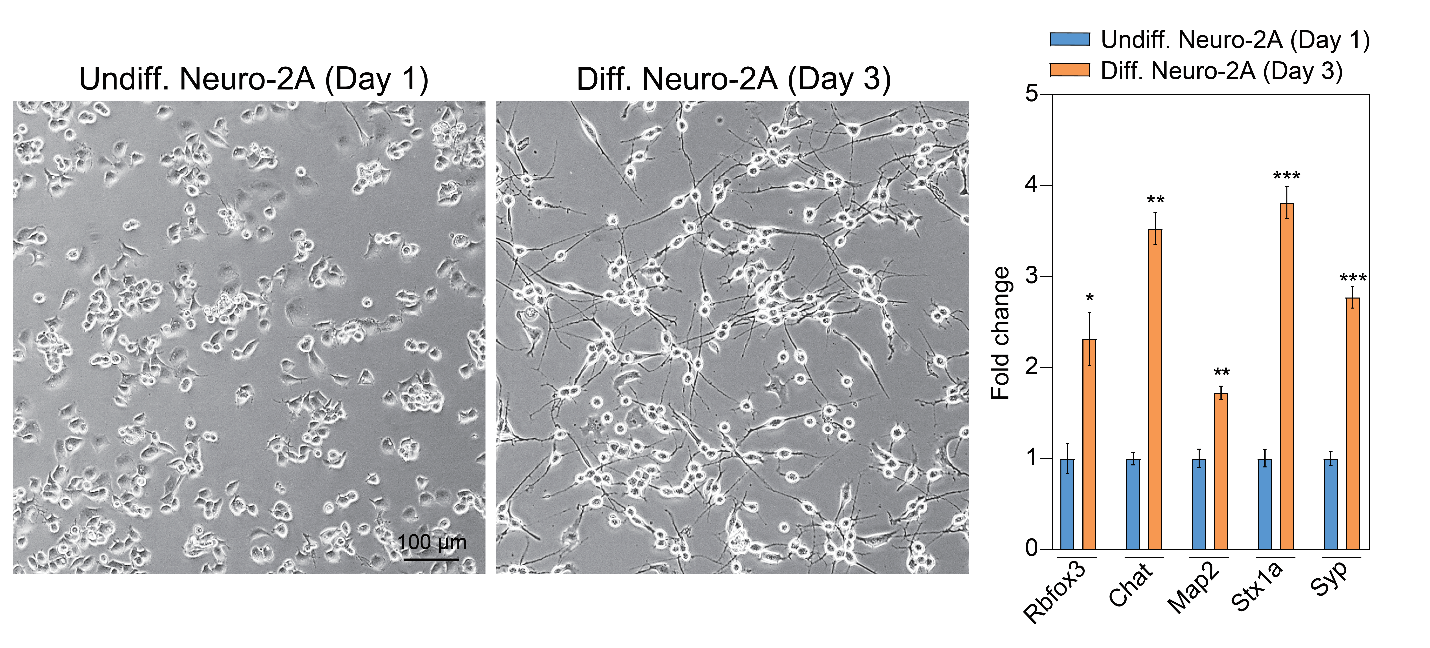


**Supplementary Fig. S3**


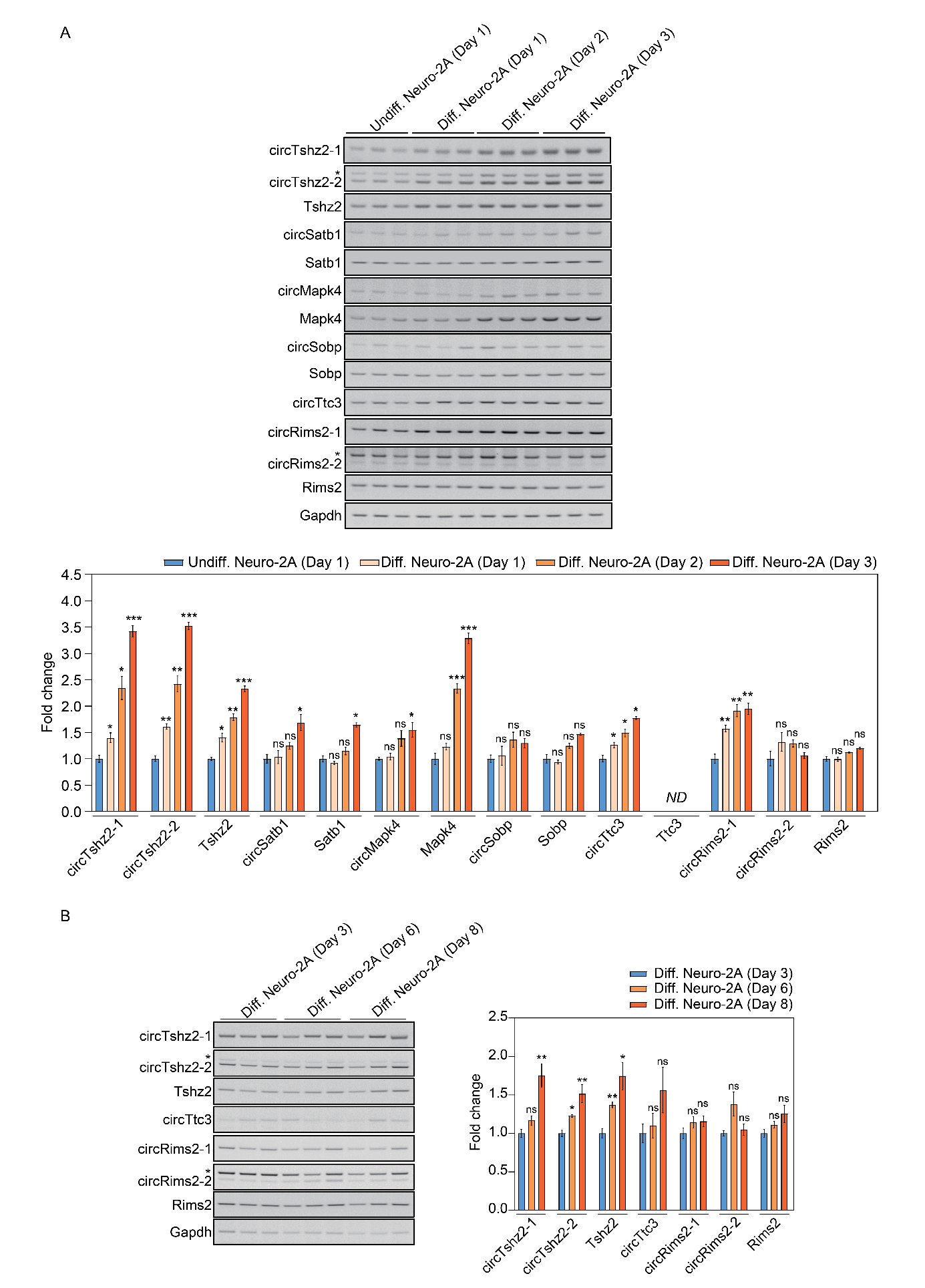


**Supplementary Fig. S4**


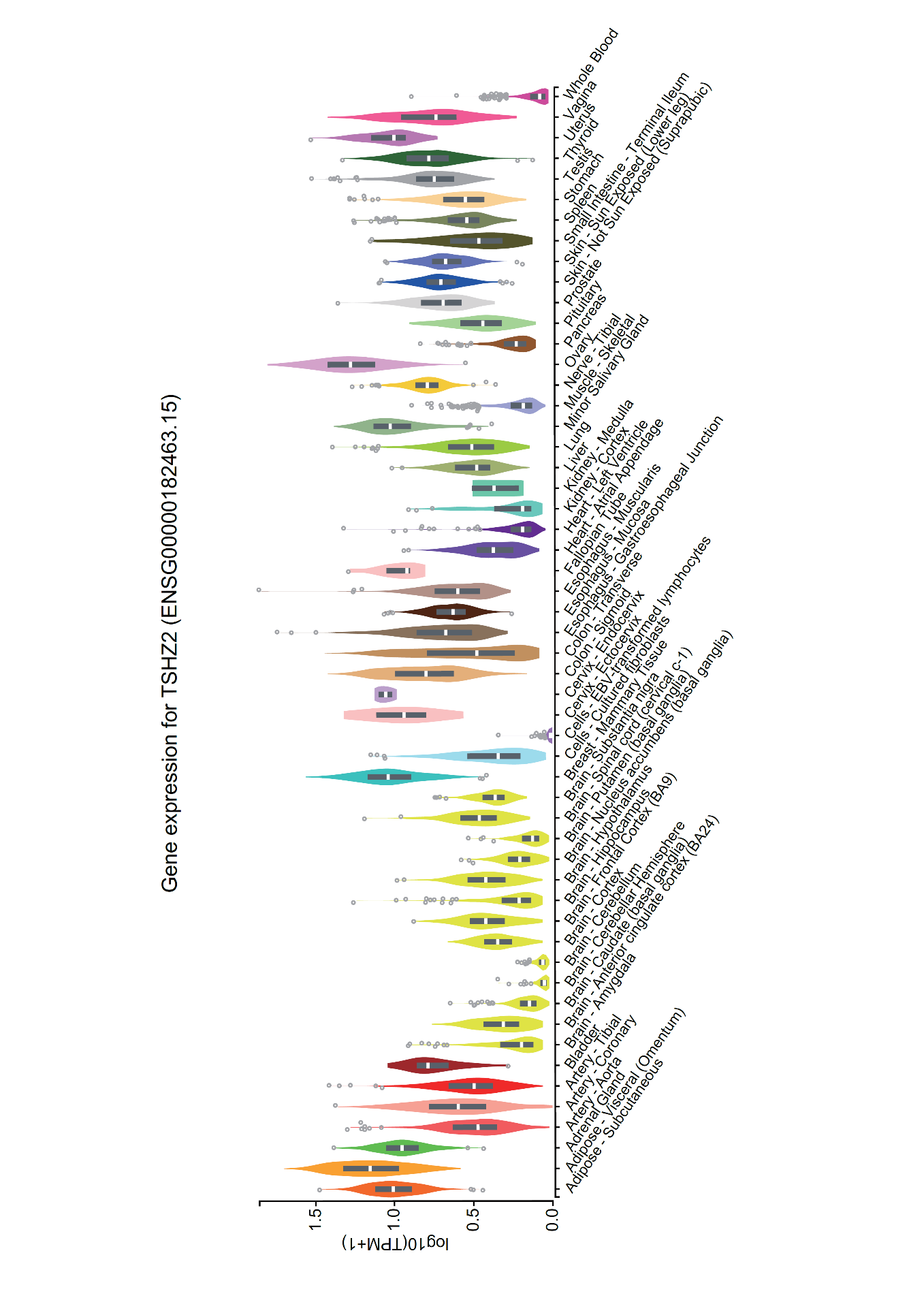


**Supplementary Fig. S5**


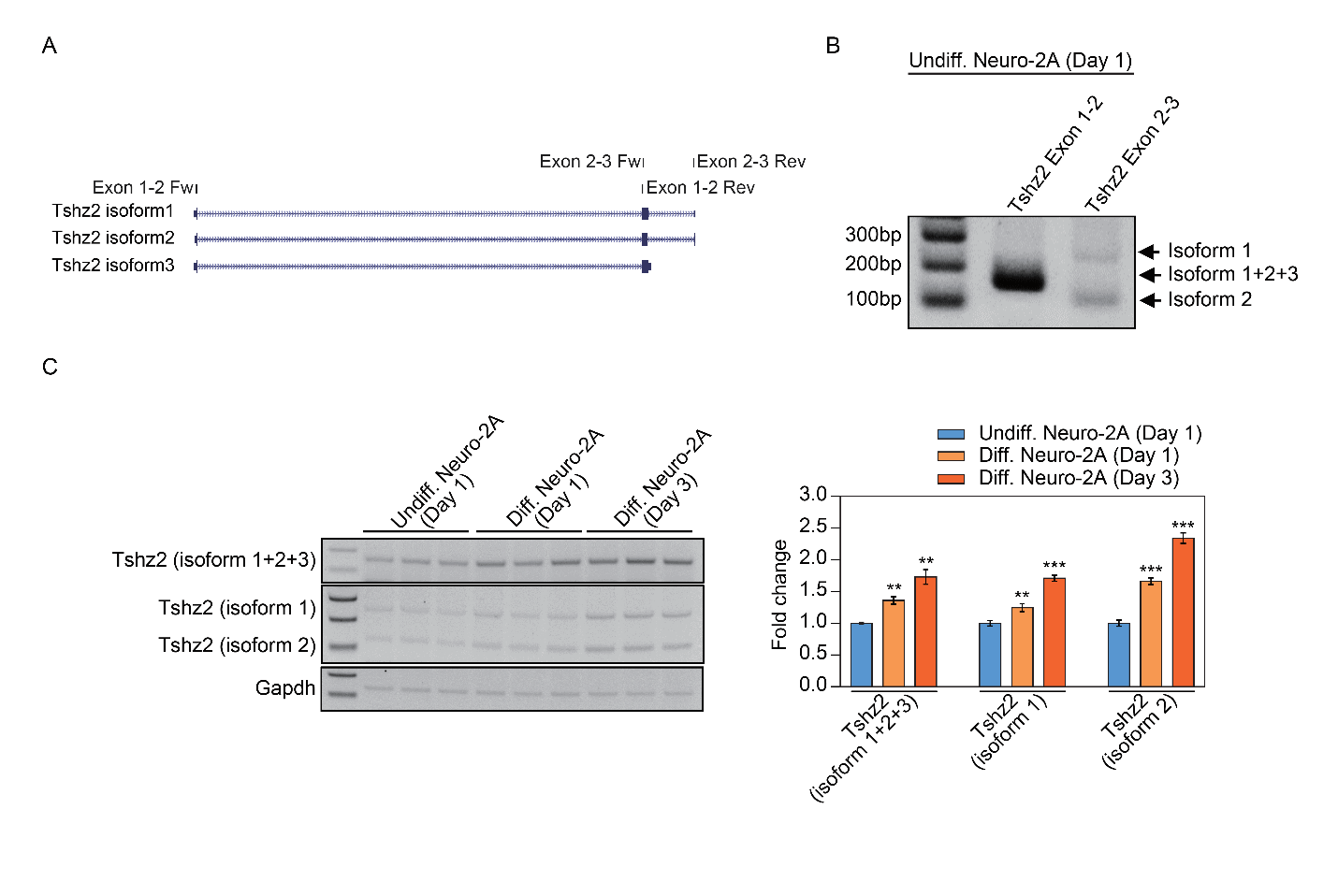


**Supplementary Fig. S6**


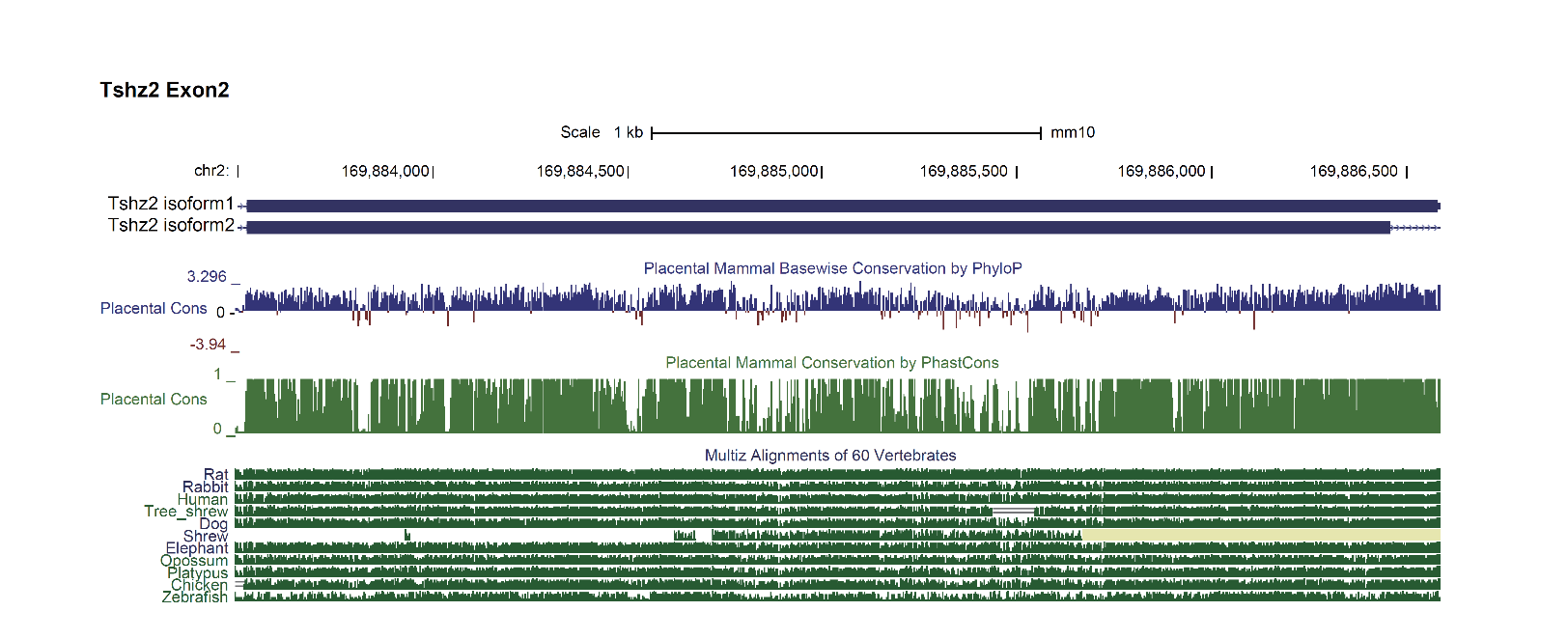


**Supplementary Fig. S7**


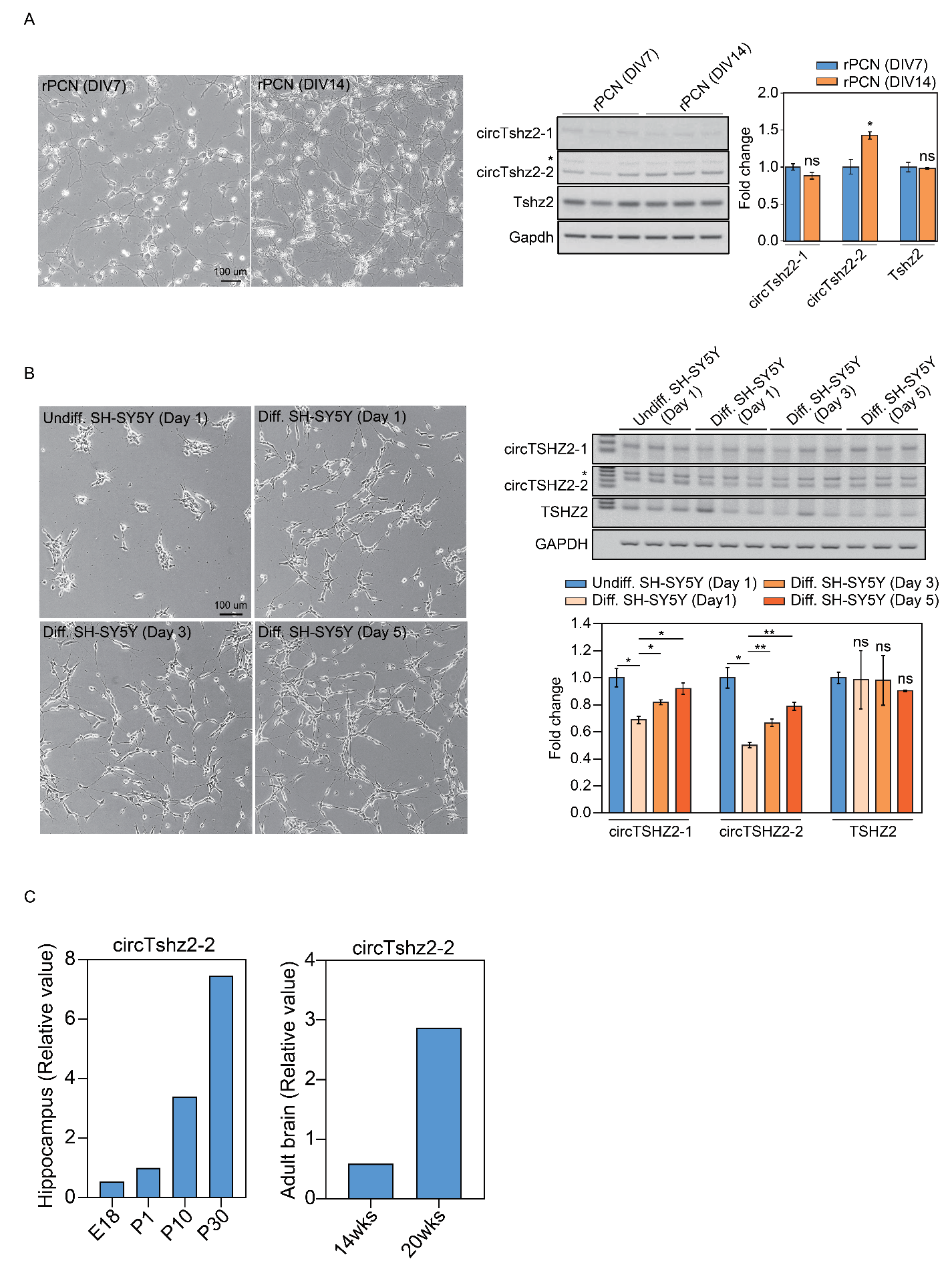


**Supplementary Fig. S8**


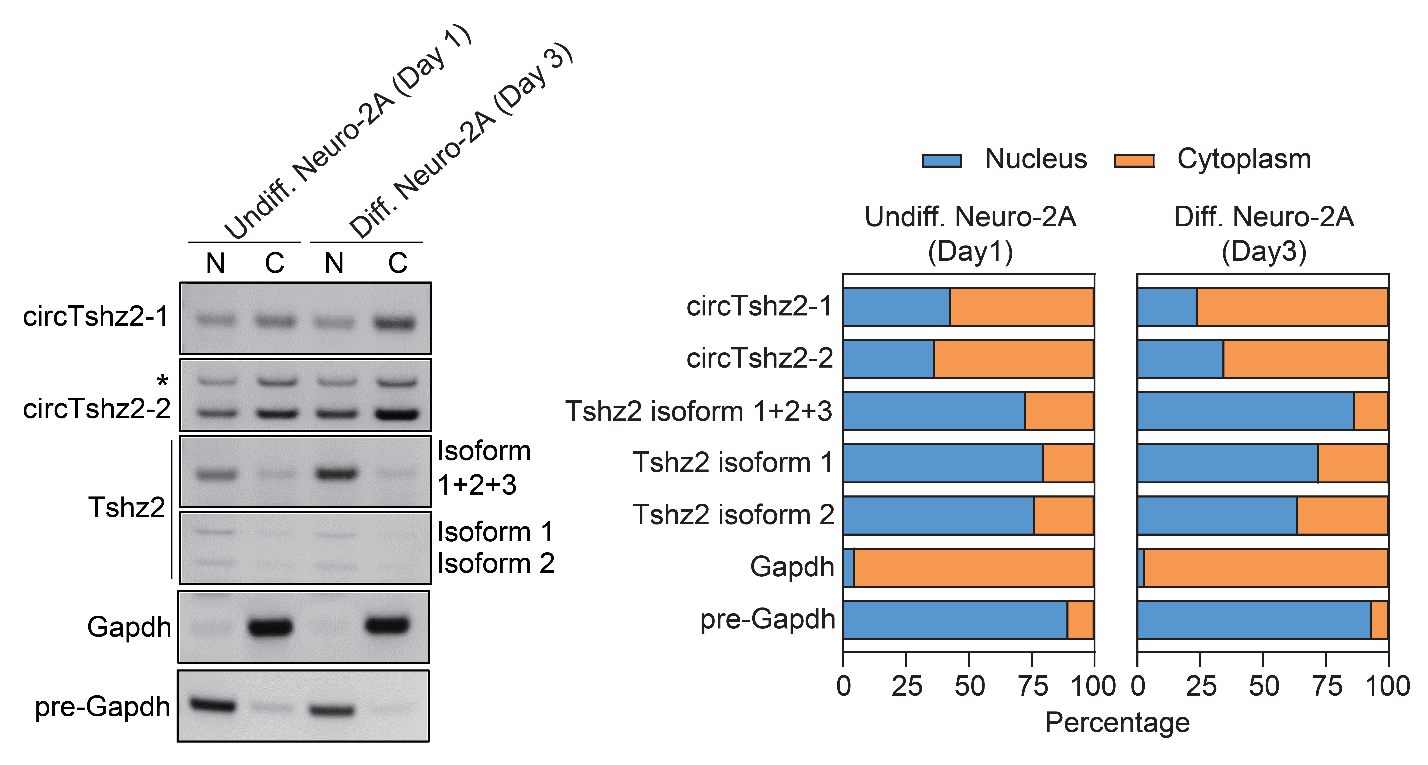


**Supplementary Fig. S9**


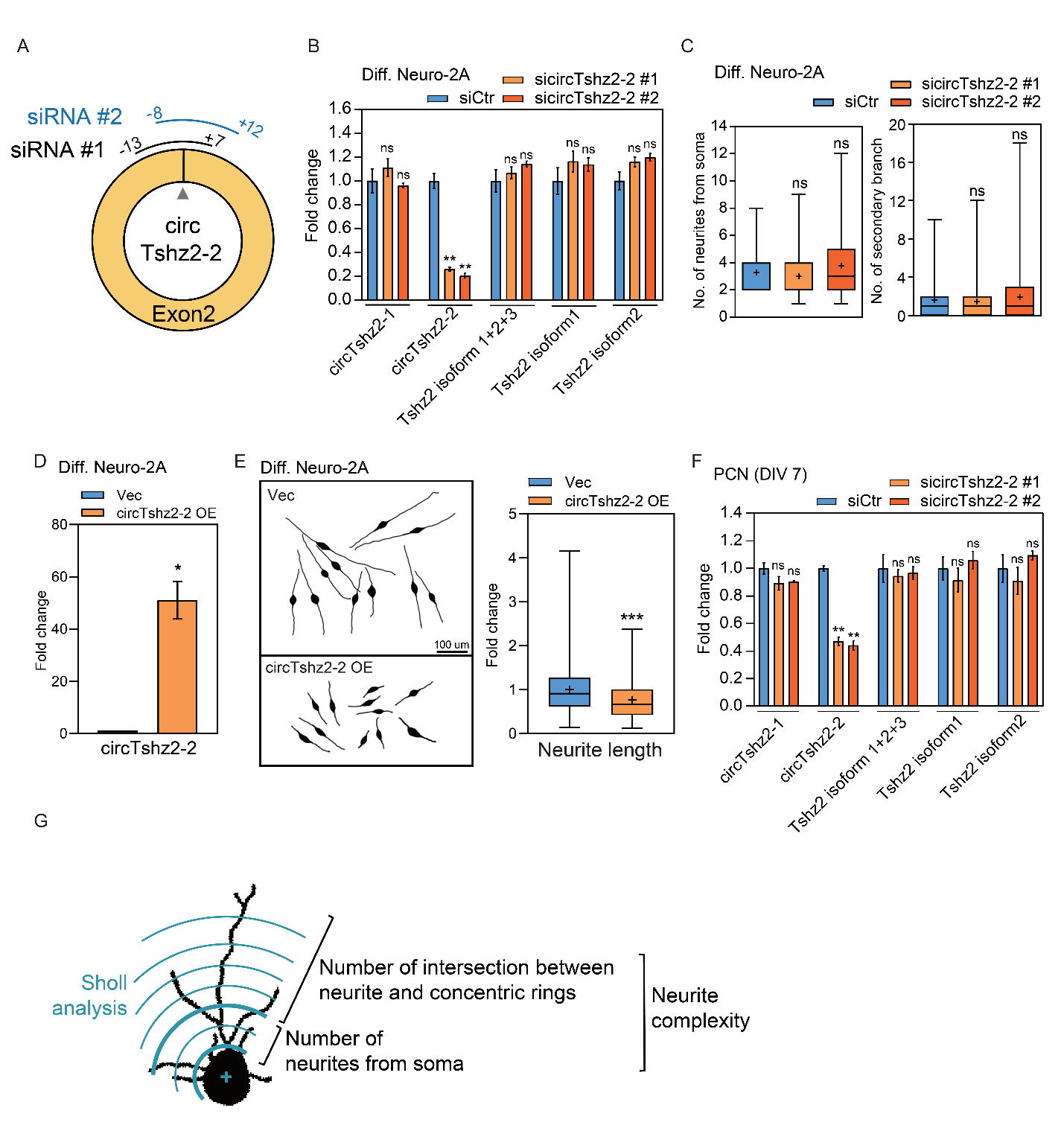


**Supplementary Fig. S10**


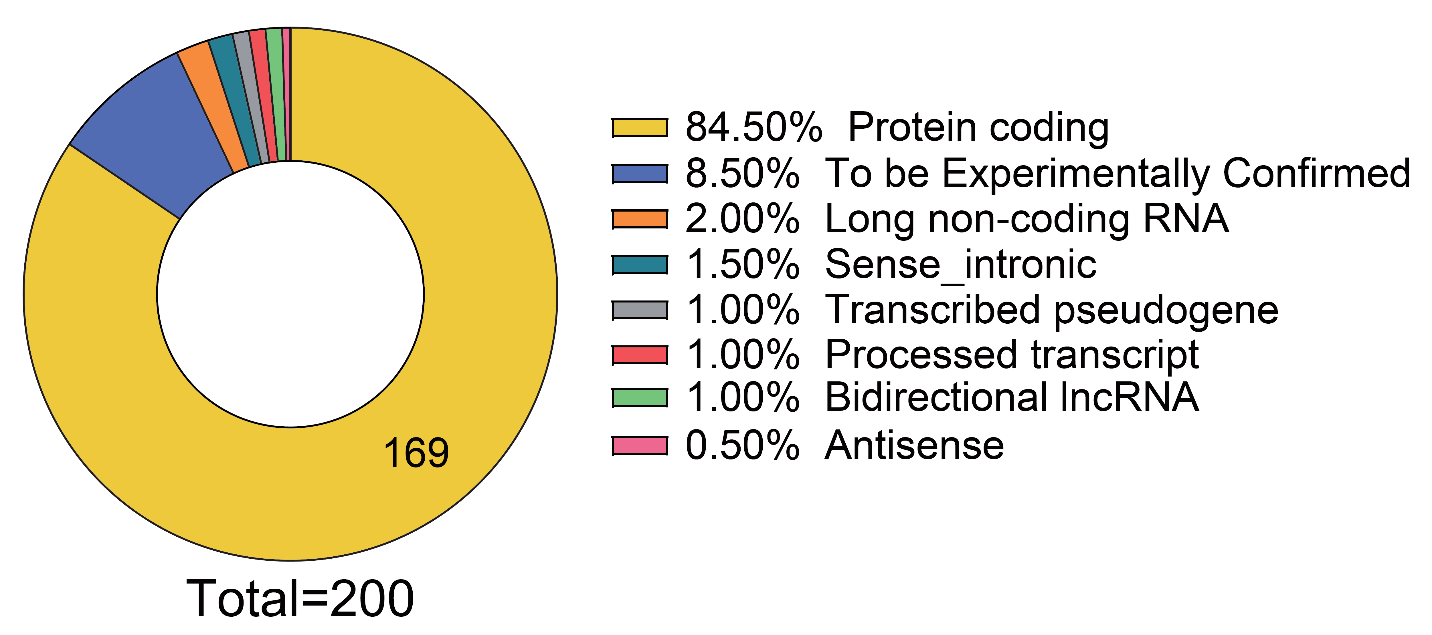


**Supplementary Fig. S11**


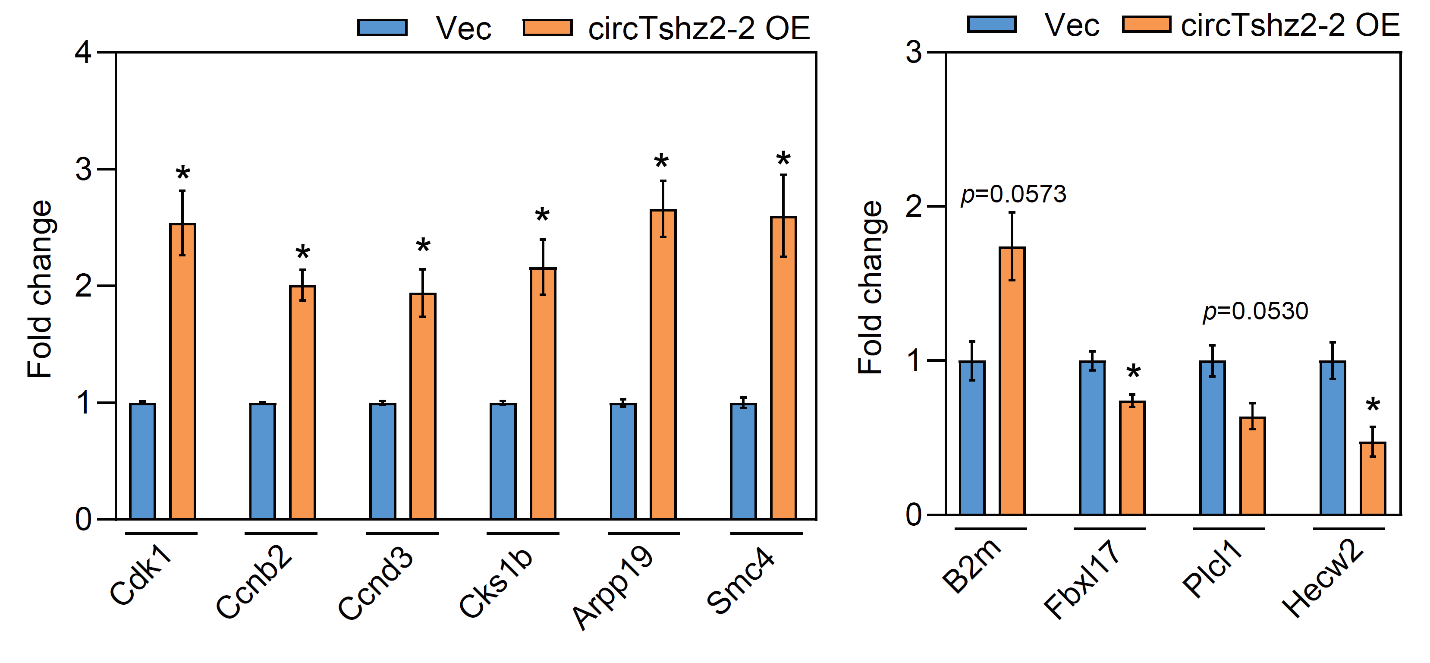


**Supplementary Fig. S12**


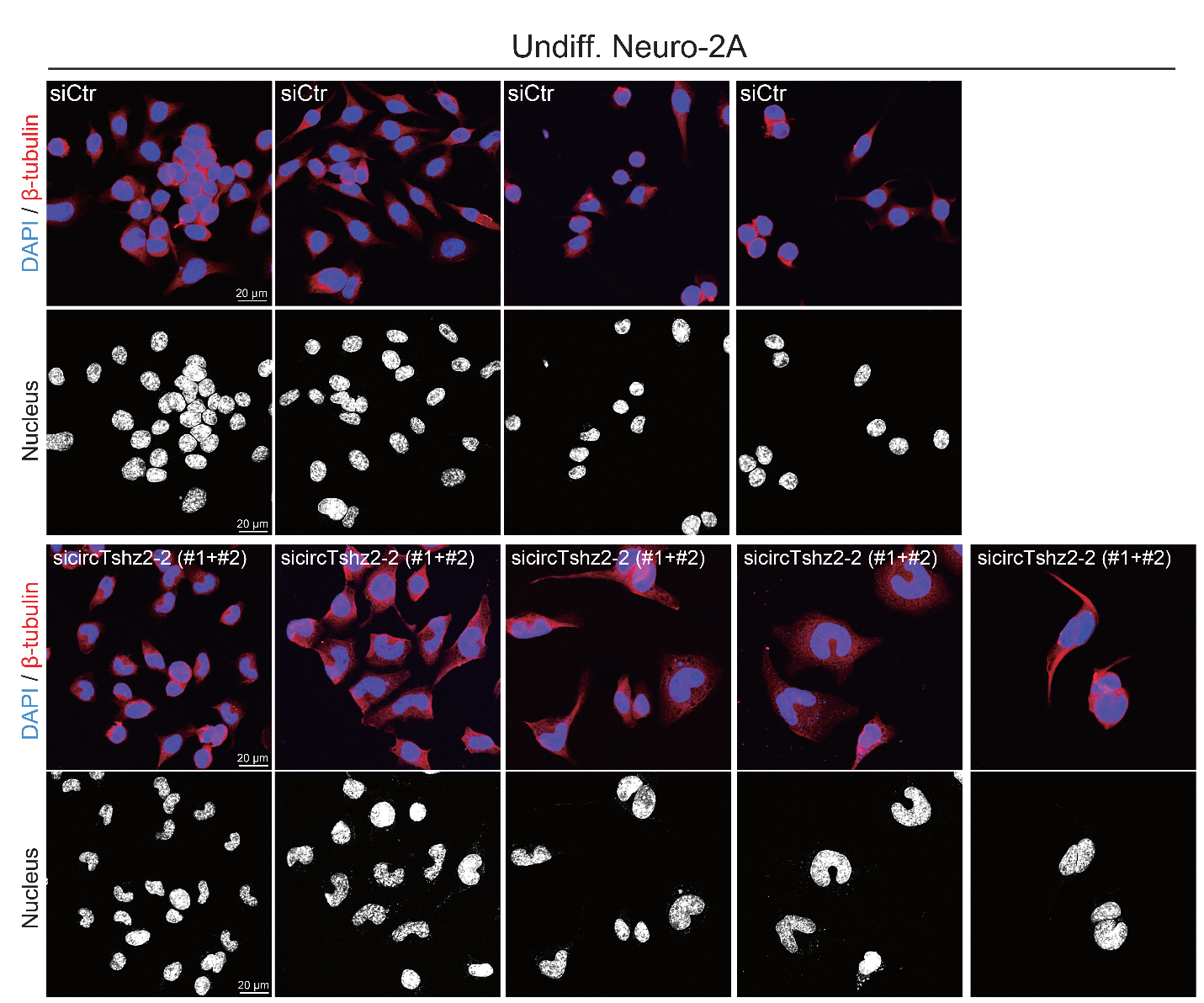


**Supplementary Fig. S13**


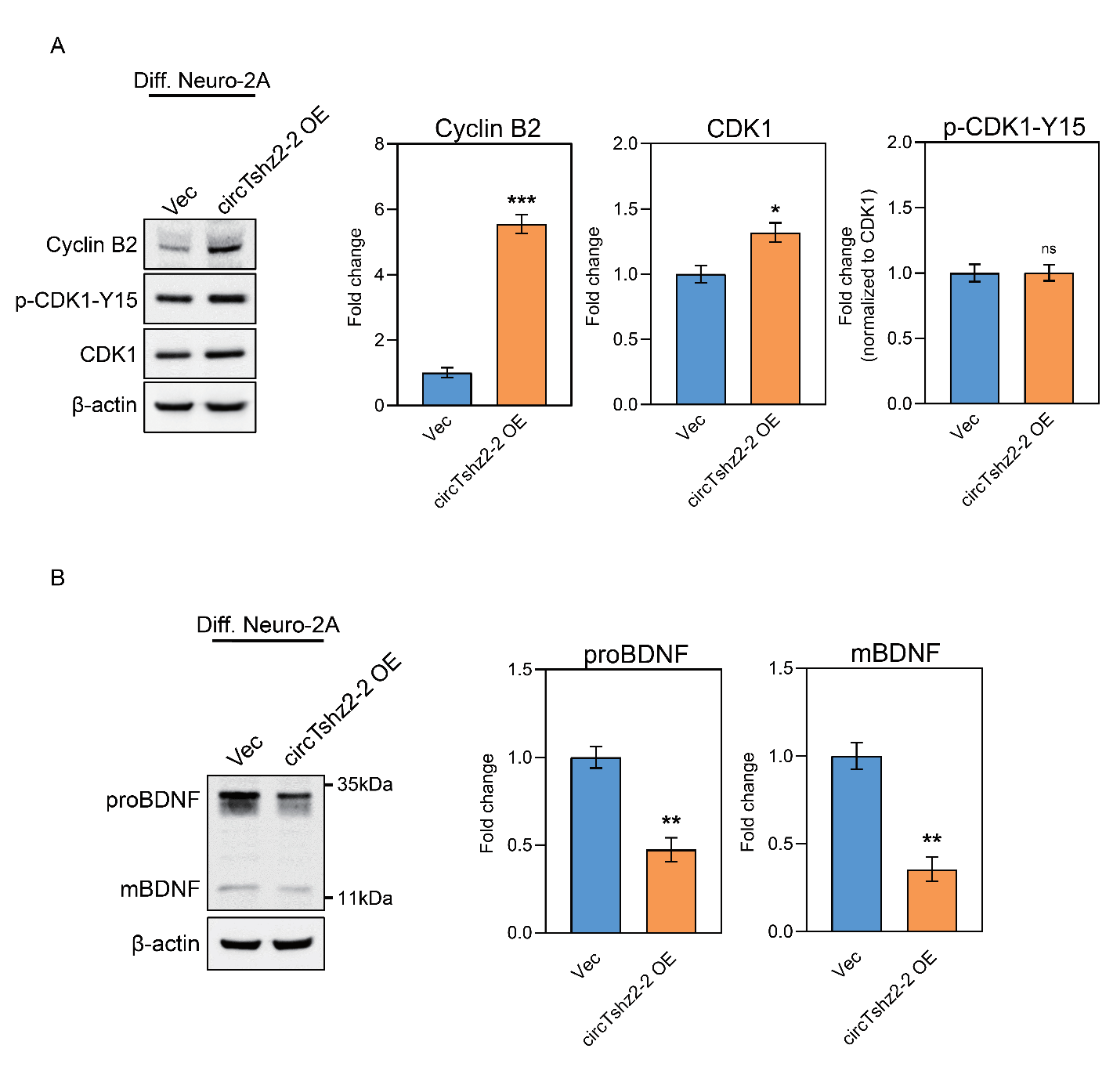


**Supplementary Fig. S14**


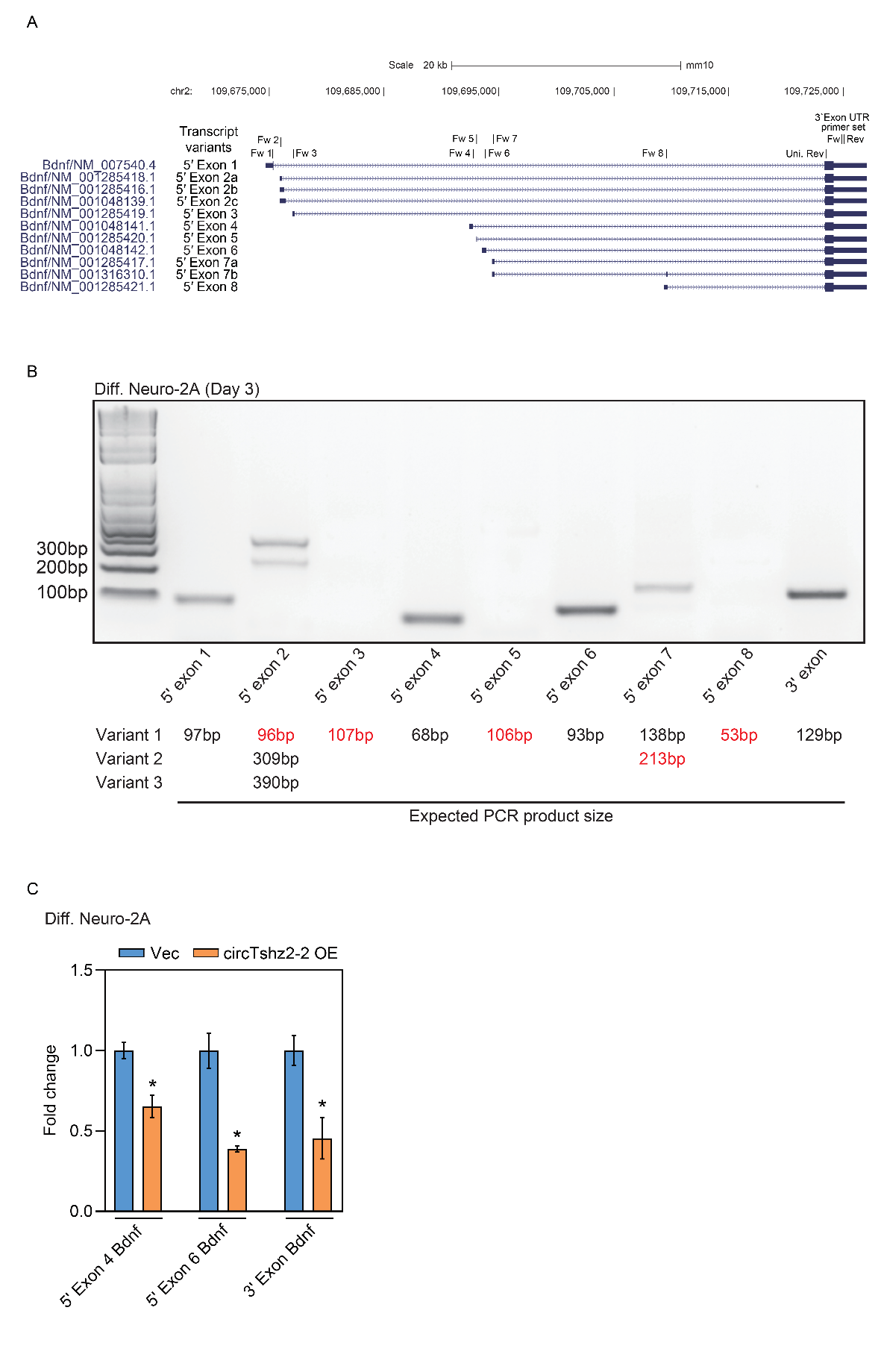


**Supplementary Fig. S15**


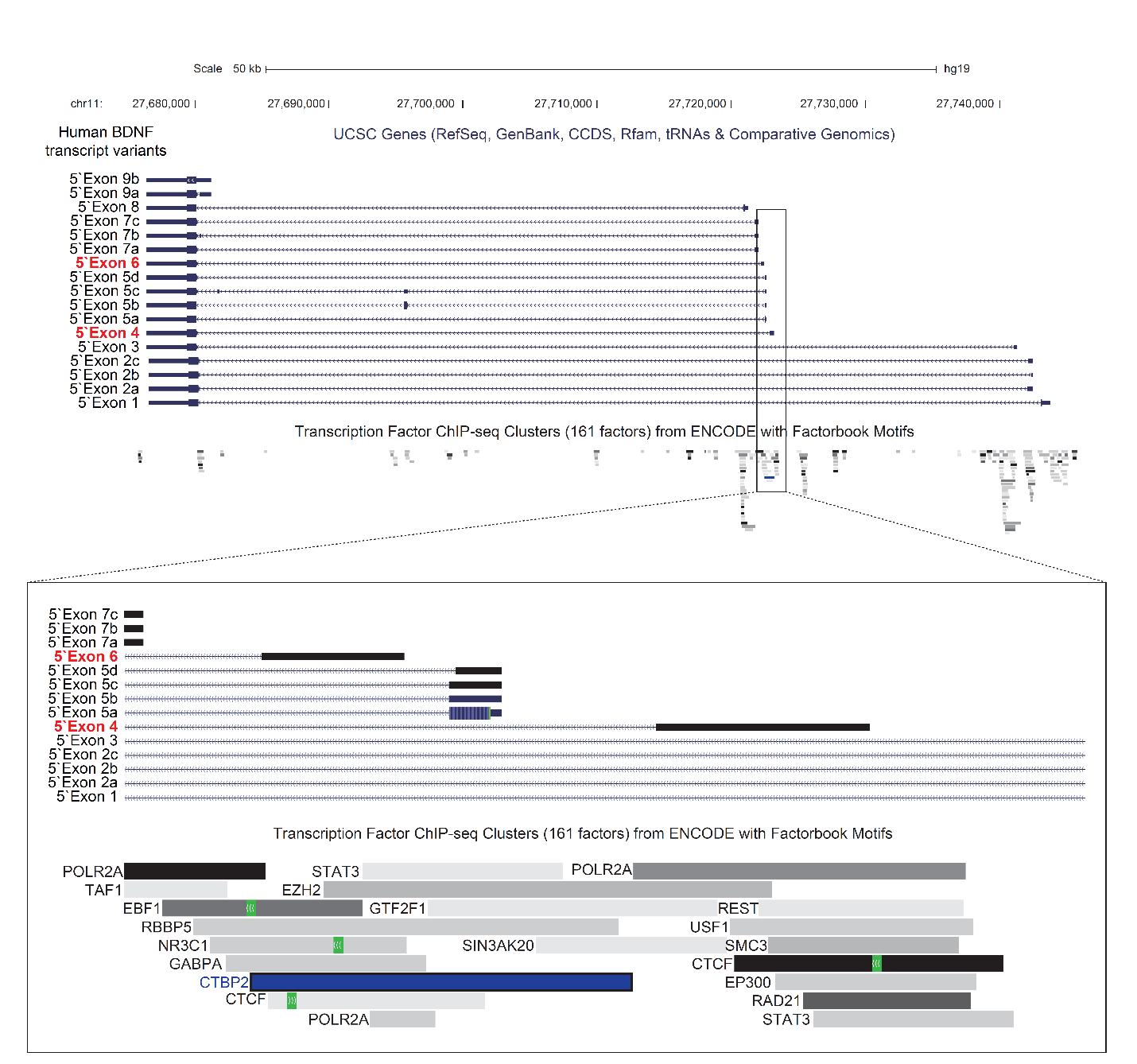


**Supplementary Fig. S16**


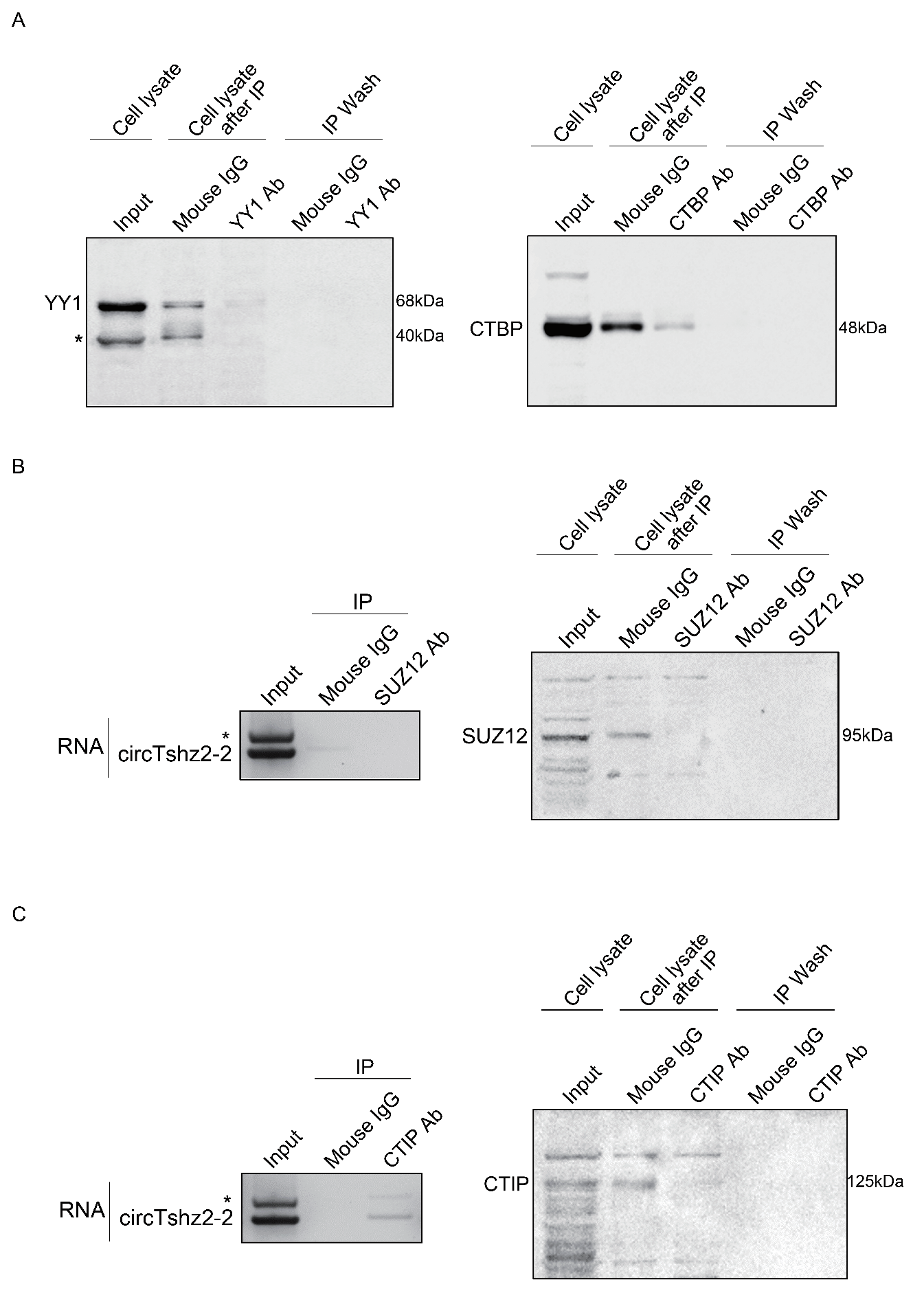


**Supplementary Fig. S17**


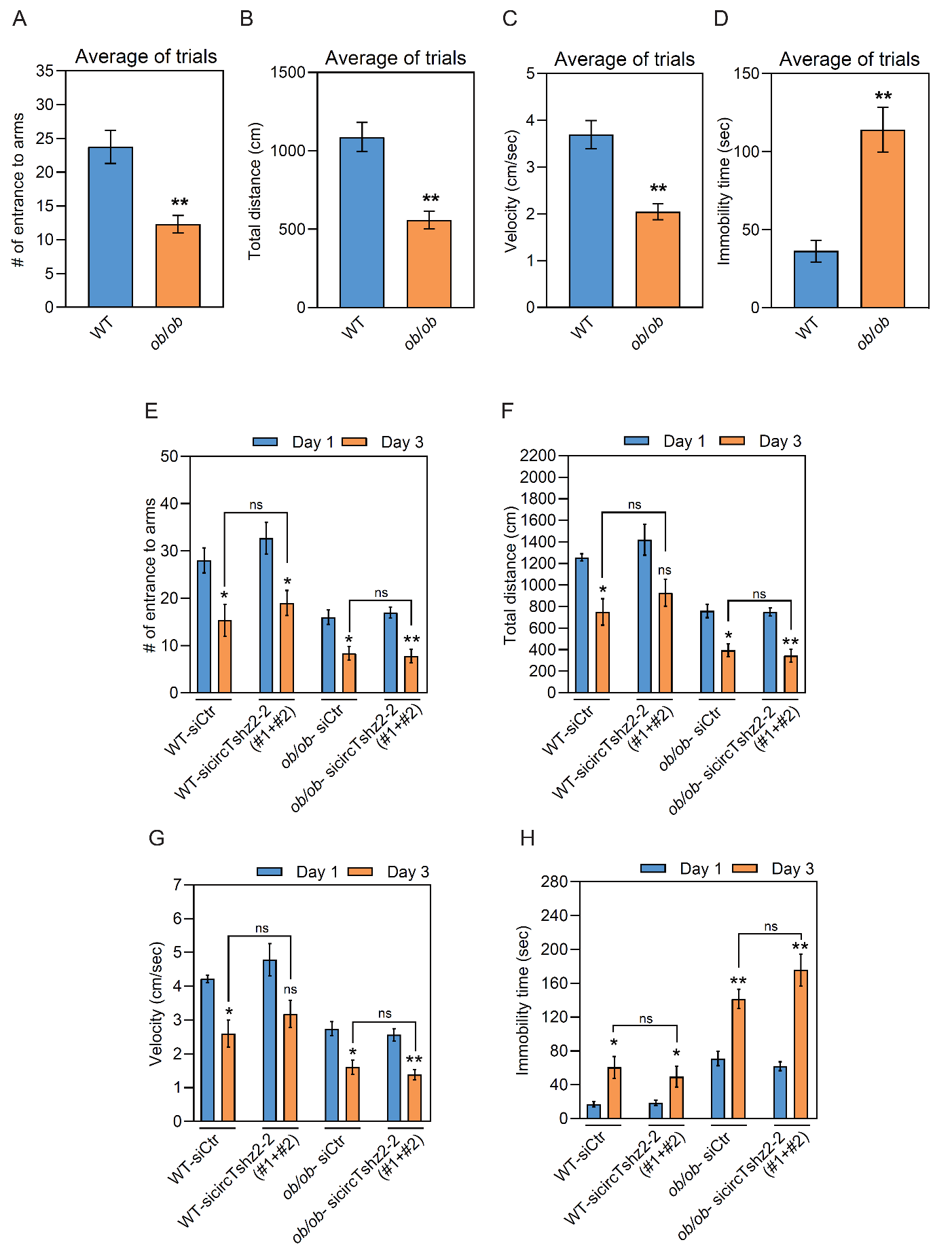


**Supplementary Fig. S18**


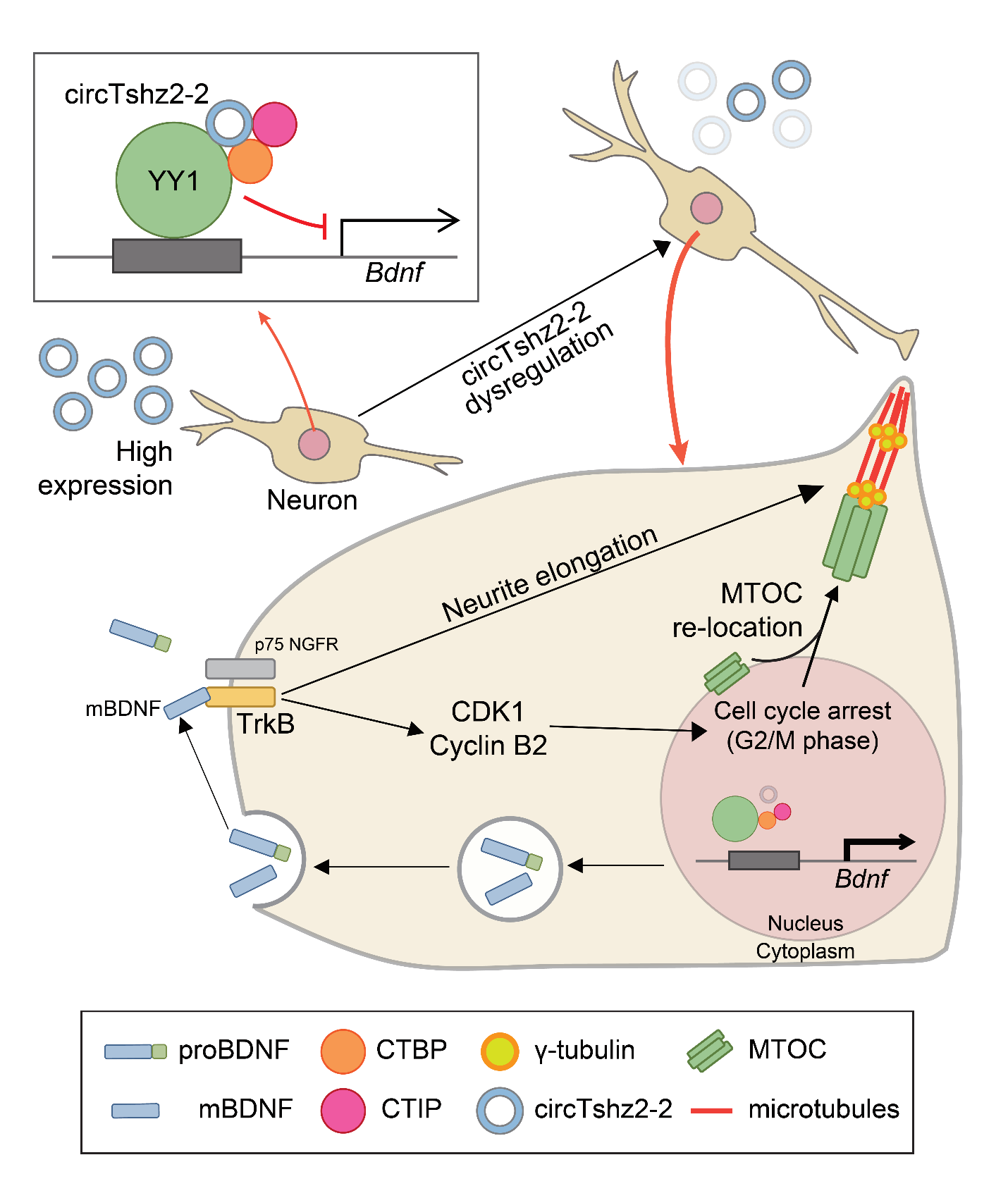


**Supplementary Figure Legends**

Supplementary Fig. S1.

**Cell type-specific expression of obesity-related circRNAs in the brain.**The specific expression of obesity-related circRNAs in microglial (BV-2), astrocytic (C8-D1a), and neuronal cells (Neuro-2A). This expression was measured as the mean of three samples (n=3) and depicted as a relative percentage in each cell type. The expected PCR product size for each circRNA is shown in the parentheses. The band marked with an asterisk indicates another isoform of each circRNA.

Supplementary Fig. S2.

**Verification of Neuro-2A neuronal differentiation.**The changes in cell morphology and gene expression associated with neuronal differentiation and synaptic vesicle development after three days of differentiation (Diff.) in Neuro-2A cells. The cell morphology was evaluated in three independent cell cultures (n=3) and the markers of neuronal differentiation (*Rbfox3*, *Chat*, *Map2*) and synaptic vesicle development (*Stx1a* and *Syp*) are reported as the mean ± SEM (n=3). "Undiff." indicates undifferentiated cells and statistical significance was analyzed using an unpaired *t-*test with Welch's correction; *: *p*<0.05, **: *p*<0.01, ***: *p*<0.001.

Supplementary Fig. S3.

**Differential expression of obesity-related circRNAs during neuronal differentiation. (A)** Changes in the expression of each circRNA during neuronal differentiation are described as the mean ± SEM (n=3). Undiff., Diff., and ND indicate undifferentiated, differentiated, and not determined, respectively. **(B)** Changes in the expression of each circRNA during neuronal differentiation post day 3 are described as the mean ± SEM (n=3). In **(A)** and **(B)**, the band marked with an asterisk indicates another isoform of each circRNA, and statistical significance was evaluated using an unpaired *t-*test with Welch's correction; ns: not significant, *: *p*<0.05, **: *p*<0.01, ***: *p*<0.001.

Supplementary Fig. S4.

**The Genotype-Tissue Expression (GTEx) data for human TSHZ2.**The median gene expression levels of TSHZ2 in 52 human tissues and 2 human cell lines, based on RNA-seq data from the GTEx final data release V8. Each tissue was assigned its color following the GTEx consortium publication conventions. The data are shown as log_10_(Transcripts Per Million + a pseudo count number 1).

Supplementary Fig. S5.

**The expression of mouse Tshz2 isoforms during neuronal differentiation. (A)** Genomic context of the Tshz2 isoforms obtained from the UCSC Genome Browser with each of the primer sets used to distinguish each Tshz2 isoform. Fw and Rev indicate the forward and reverse primers, respectively. **(B)** The expression of each Tshz2 isoform in undifferentiated (Undiff.) Neuro-2A cells at day 1. The data are from a representative experiment from three replicates with similar results (n=3). **(C)** The changes in the expression of each Tshz2 isoform during neuronal differentiation. The data are described as the mean ± SEM (n=3). "Diff." indicates differentiated and statistical significance was determined using an unpaired *t-*test with Welch's correction; **: *p*<0.01 and ***: *p*<0.001.

Supplementary Fig. S6.

**The genomic context for *Tshz2* exon 2.**The genomic context and preservation of *Tshz2* exon 2 in various species. The genomic context and degree of conservation of these genes in placental mammals (PhyloP and PhastCons) were obtained from the UCSC Genome Browser. *Tshz2* exon 2 is evolving more slowly than expected (positive score in PhyloP) and is well conserved in mammals (closer to 1 in PhastCons). Multiz Alignments of 60 vertebrates show the high degree of conservation of the *Tshz2* exon 2 across species (99.6% of bases in human, 99.8% of bases in rat, 99.7% of bases in rabbit, 99.5% of bases in rhesus, 98.8% of bases in chicken, 99.9% of bases in dog, 98.1% of bases in frog, and 87.1% of bases in zebrafish).

Supplementary Fig. S7.

**Expression of circTshz2-1, circTshz2-2, and Tshz2 during neuronal differentiation, maturation, and nervous system development across species. (A)** Changes in cell morphology and expression of circTshz2-1, circTshz2-2, and Tshz2 during rat primary cortical neuron (rPCN)’s maturation. Cell morphology data is representative of three independent cell cultures, and gene expression data is described as the mean ± SEM (n=3). DIV indicates the days *in vitro*. **(B)** Changes in cell morphology and differential expression of circTSHZ2-1, circTSHZ2-2, and TSHZ2 during human SH-SY5Y neuronal differentiation. "Undiff." and "Diff." indicate undifferentiated and differentiated cells, respectively. The cell morphology data is representative of three independent cell cultures, and the gene expression data is reported as the mean ± SEM (n=3). In **(A)** and **(B)**, the band marked with an asterisk is circTshz2-1. **(C)** The expression of circTshz2-2 during mouse hippocampal development and adult mouse brain development was obtained from the Gene Expression Omnibus (GEO) dataset. E, P, and wks. indicate embryonic day, postnatal day, and weeks, respectively. In **(A)** and **(B)**, statistical significance was determined using an unpaired *t-*test with Welch's correction; ns: not significant, *: *p*<0.05, **: *p*<0.01.

Supplementary Fig. S8.

**Intracellular localization of circTshz2-1, circTshz2-2, and Tshz2 isoforms in the neuron.**The distribution of circTshz2-1, circTshz2-2, and Tshz2 isoforms in the nucleus (N) and cytoplasm (C) of undifferentiated (Undiff.) and differentiated (Diff.) Neuro-2A cells, respectively. The average expression from three independent experiments was measured and the differences in relative expression between the nucleus and cytoplasm are shown as percentages. The band marked with an asterisk is circTshz2-1.

Supplementary Fig. S9.

**The knockdown and overexpression efficiency of circTshz2-2 and the analysis of neurite complexity. (A)** Illustration displaying the binding sites of two independent siRNAs (#1 and #2) against the back-splicing junction of circTshz2-2. The position of the nucleotides binding each siRNA is depicted as “+” or “-” relative to the back-splicing junction, which is denoted as “0”. The triangle indicates the back-splicing junction of circTshz2-2. **(B)** Changes in the expression of circTshz2-1, circTshz2-2, and Tshz2 isoforms following circTshz2-2 knockdown in differentiated (Diff.) Neuro-2A cells at day 5 are described as the mean ± SEM (n=3). siCtr indicates negative control siRNA. **(C)** Changes in the number of neurites from the soma and secondary branching following circTshz2-2 knockdown in differentiated (Diff.) Neuro-2A cells at day 5. **(D)** Changes in the expression of circTshz2-2 following its overexpression (OE) in differentiated (Diff.) Neuro-2A cells at day 5 are described as the mean ± SEM (n=3). Vec indicates the control vector. **(E)** Changes in neural structures and total neurite length following circTshz2-2 overexpression (OE) in differentiated (Diff.) Neuro-2A cells at day 5. Vec indicates the control vector. **(F)** Changes in the expression of circTshz2-1, circTshz2-2, and Tshz2 isoforms following circTshz2-2 knockdown in mouse primary cortical neurons (PCN) at 7 days *in vitro* (DIV 7) are described as the mean ± SEM (n=3). **(G)** Illustration describing the methods used to analyze the number of neurites from each soma and the number of intersections using Sholl analysis. The circular arc (blue-green) is a constant interval of concentric rings used in the Sholl analysis. In **(C)** and **(E)**, the whiskers in graphs represent the minimum and maximum values while the box line represents the first quartile, median, and third quartile, respectively. The "+" indicates the mean. In **(C)** and **(E)**, structural data represents the findings from at least three independent experiments (n=3) with more than 10 neurons analyzed per replicate. In **(B)** through **(F)**, statistical significance was determined using an unpaired two-tailed *t-*test with Welch's correction; ns: not significant, *: *p*<0.05, **: *p*<0.01, ***: *p*<0.001.

Supplementary Fig. S10.

**Gene types of circTshz2-2 responsive genes.**Illustration showing the percentage of gene types for the 200 differentially expressed genes identified during the circTshz2-2 knockdown. The gene type categories are based on the GENCODE annotation.

Supplementary Fig. S11.

**Genes altered by circTshz2-2 overexpression in differentiated Neuro-2A cells.**The expression changes of genes involved in cell cycle regulation, chromosome segregation, and neuronal function following circTshz2-2 overexpression (OE) were confirmed in the differentiated Neuro-2A cells at day 5 and depicted as the mean ± SEM (n=3). Vec indicates the control vector. The statistical significance was analyzed using an unpaired two-tailed *t*-test with Welch's correction; *: *p*<0.05.

Supplementary Fig. S12.

**Nuclear morphology of undifferentiated Neuro-2A cells after the circTshz2-2 knockdown.**The change in nuclear morphology following circTshz2-2 knockdown in undifferentiated (Undiff.) Neuro-2A cells at day 5. This data is depicted using representative cells from three independent cultures (n=3), and the beta-tubulin and nuclei are indicated by red and blue colors, respectively. siCtr indicates negative control siRNA. The data from sicircTshz2-2-treated cells was made by treating the cells with siRNA mixtures of two different siRNAs against circTshz2-2 (#1 and #2).

Supplementary Fig. S13.

**The expression of cell cycle proteins and BDNF after circTshz2-2 overexpression in differentiated Neuro-2A cells. (A)** The changes in the protein levels of Cyclin B2, CDK1, and p-CDK1-Y15 following circTshz2-2 overexpression (OE) in differentiated (Diff.) Neuro-2A cells at day 5. Vec indicates the control vector. p-CDK1-Y15 indicates the CDK1 protein phosphorylated at tyrosine 15. The level of p-CDK1-Y15 was normalized against CDK1. **(B)** The change in the protein levels of precursor BDNF (proBDNF) and mature BDNF (mBDNF) following circTshz2-2 overexpression (OE) in differentiated (Diff.) Neuro-2A cells at day 5. Vec indicates the control vector. In **(A)** and **(B)**, the expression changes are depicted as the mean ± SEM (n=3), and the statistical significance was analyzed using an unpaired two-tailed *t-*test with Welch's correction; ns: not significant, *: *p*<0.05, **: *p*<0.01, ***: *p*<0.001.

Supplementary Fig. S14.

**Genomic context of mouse Bdnf and its expression change by circTshz2-2 overexpression. (A)** Genomic context of the 11 mouse Bdnf transcript variants identified at the UCSC Genome Browser. The eight forward (Fw) primers, a universal reverse (Uni. Rev) primer, and a primer set for the 3′ untranslated region (UTR) are shown. **(B)** Confirmation of Bdnf transcript variant expression in differentiated (Diff.) Neuro-2A cells at day 3. This data was selected from a representative experiment among a triplicate with similar results (n=3). The expected sizes of PCR products for each Bdnf isoform are shown, and undetected isoforms are marked in red. **(C)** The change in the level of Bdnf isoforms following circTshz2-2 overexpression (OE) in differentiated (Diff.) Neuro-2A cells at day 5. The data is depicted as the mean ± SEM (n=3). Vec indicates the control vector. The statistical significance was analyzed using an unpaired two-tailed *t-*test with Welch's correction; *: *p*<0.05.

Supplementary Fig. S15.

**Possible transcriptional regulators of *BDNF* with potential binding sites between exons 4 and 6 of the human *BDNF* gene.**The genomic information for the human *BDNF* gene was obtained from the UCSC Genome Browser. The cluster of possible transcription factors obtained from ChIP-seq data in the ENCODE project is displayed. The BDNF transcript variants between exons 4 and 6 are magnified and annotated with possible transcriptional regulators. CTBP2, a TSHZ2 co-factor, is marked in blue.

Supplementary Fig. S16.

**RNA-binding protein immunoprecipitation (RNA-IP). (A)** The RNA-IP experiments were designed to confirm whether each antibody used in this experiment efficiently bound to target proteins (YY1 and CTBP). This data was selected from a representative experiment among triplicates with similar results (n=3). The “cell lysate after IP” represents the residual cell lysate after being immunoprecipitated with magnetic beads and antibody complexes. The “IP wash” describes the supernatant after washing the magnetic bead/protein of interest/antibody immunoprecipitates. The band marked with an asterisk indicates the cleaved form of the YY1 protein. **(B)** Interaction between circTshz2-2 and SUZ12. This data was selected from a representative experiment among triplicates with similar results (n=3). The band marked with an asterisk is circTshz2-1. IP indicates immunoprecipitation. **(C)** The RNA-protein interactions between circTshz2-2 and CTIP. This data was selected from a representative experiment among triplicates with similar results (n=3). The band marked with an asterisk is circTshz2-1.

Supplementary Fig. S17.

**The locomotor activities of wild-type and obese mice. (A)** The average number of the entrance to the arms, **(B)** the average total distance (cm), **(C)** the average velocity (cm/sec), and **(D)** the average immobility time (sec), for wild-type (WT) and obese (*ob*/*ob*) mice, respectively. In **(A)** through **(D)**, the data were obtained from day 1 and day 3 during trials to calculate the average value. **(E)** The changes in the number of the entrance to the arms, **(F)** the changes in the total distance (cm), **(G)** the changes in the velocity (cm/sec), and **(H)** the changes in the immobility time (sec), from day 1 to 3, following infusion of either control siRNA or circTshz2-2 siRNAs into wild-type and obese mice were presented, respectively. In **(A)** through **(H)**, the data are depicted as the mean ± SEM (n=5). In **(E)** through **(H)**, WT-siCtr indicates control siRNA-infused wild-type mouse, and WT-sicircTshz2-2 (#1+#2) indicates the wild-type mice infused with two circTshz2-2 siRNAs. ob/ob-siCtr indicates control siRNA-infused obese mice. ob/ob-sicircTshz2-2 (#1+#2) indicates the obese mice infused with two circTshz2-2 siRNAs. Statistical significance for **(A)** through **(H)** was determined using an unpaired two-tailed *t*-test with Welch’s correction; ns: not significant, *: *p*<0.05, **: *p*<0.01.

Supplementary Fig. S18.

**The proposed mechanism of action for circTshz2-2 in the neuron.**Highly expressed circTshz2-2 binds to the YY1 transcriptional repressor complex, resulting in the repression of *Bdnf* transcription in the neuron (upper left). When the circTshz2-2 expression is suppressed, *Bdnf* transcription is initiated, increasing the levels of precursor BDNF (proBDNF) and mature BDNF (mBDNF). This increase allows for more mBDNF interaction with the tyrosine kinase B (TrkB) receptor and the inactivation of the Cyclin B2/CDK1 complex. This inactivation then results in G2/M cell cycle arrest. This G2/M phase arrest re-locates the microtubule-organizing center (MTOC) to the neurite, increasing neurite length and complexity in concert with BDNF/TrkB receptor activation.

**Supplementary Table Legends**

The supplementary table files are available online.

Supplementary Table S1.

**The list of siRNAs and PCR primers.** The sequences of two independent siRNAs (#1 and #2) of circTshz2-2 are included. The sequences (mouse) of forward and reverse primer for Bdnf transcript variants, those genes related to cell cycle, neuronal function, and neuronal differentiation, and the sequences of circRNA candidates and their host genes are included. The sequences (rat and human) of forward and reverse primer for circTshz2-1, circTshz2-2, and Tshz2 are also included.

Supplementary Table S2.

**The list of primers used for circRNA cloning.** The sequences of primer set for the cloning of circTshz2-2 are listed. The primers to amplify circTshz2-2 fragments, and the primers containing the restriction enzyme site for the insertion into the vector are included. The primers to verify the circTshz2-2 overexpression vector are also included.

Supplementary Table S3.

**The data from bioinformatics analyses.** The biological process terms of Gene Ontology (GO) analysis are included in the order of significant false discovery rate (FDR) values. The predicted transcription factors and chromatin regulators are included in the order of Irwin-Hall *p*-value (BART) and Integrated Rank Score (ChEA3), respectively. The interaction probability scores between circTshz2-2 sequence (input) and predicted transcription regulators (BART and ChEA3) by the RPIseq tool are also included with the information of GENCODE transcript number and amino acid length for each protein.
