# Supplementary material for: Obesity-linked circular RNA circTshz2-2 regulates the neuronal cell cycle and spatial memory in the brain
Source: Mol Psychiatry. 2021 Sep 24;26(11):6350–64. doi: 10.1038/s41380-021-01303-x (PMC8760052; doi:10.1038/s41380-021-01303-x)
Supplement: Supplementary file 2 — Supplementary materials and methods [file 41380_2021_1303_MOESM2_ESM.docx]

Supplementary materials and methods for

**Obesity-linked circular RNA circTshz2-2 regulates the neuronal cell cycle and spatial memory in the brain**

Gwangho Yoon, Yeong-Hwan Lim, Danbi Jo, Juhee Ryu, Juhyun Song*, Young-Kook Kim*

*Corresponding author. Email: Young-Kook Kim: ykk@jnu.ac.kr; Juhyun Song: Juhyunsong@chonnam.ac.kr

**Supplementary materials and methods**

**Animal care**

Eight-week-old male wild-type C57BL/6J (Koatech) and C57BL/6J-*ob*/*ob* (Japan SLC) were housed in the Laboratory Animal Research Center, Chonnam National University (CNU), under a 16-h light/8-h dark cycle at 23⁰C with 60 humidity and given *ad libitum* access to food and water when the experimental procedures were conducted. The experiments were performed following the recommendations of the 96 guidance for animal experiments established by the Animal Ethics Committee at CNU. The Animal Ethics Committee approved the protocol at CNU. The study was carried out in compliance with the ARRIVE guidelines.

**Cell lines and culture**

Mouse Neuro-2A neuroblastoma cells, mouse BV-2 microglial cells, mouse C8-D1a astrocytic cells, and human SH-SY5Y neuroblastoma cells were purchased from the American Type Culture Collection (ATCC). The Neuro-2A and SH-SY5Y cells were cultured in Dulbecco's Modified Eagle's Medium (DMEM, WELGENE) supplemented with 10% fetal bovine serum (FBS, Millipore), 1 mM sodium pyruvate (Thermo Scientific), and 100 U/ml penicillin-streptomycin (Thermo Scientific). The BV-2 cells were cultured in DMEM containing 5% FBS and 100 U/ml penicillin-streptomycin and the C8-D1a cells were cultured in DMEM supplemented with 10% FBS and 100 U/ml penicillin-streptomycin. Cells were cultured at 37 °C and in the presence of 5% CO_2_. The medium was replaced once every two days and the cells were subcultured in multi-well plates using prewarmed 1X PBS (GENEALL) and 0.25 % trypsin (Thermo Scientific).

We induced neuronal differentiation of the Neuro-2A and SH-SY5Y cells by adding 20 μM *all-trans* retinoic acid (Sigma Aldrich) to the DMEM supplemented with 2% FBS, 1mM sodium pyruvate, and 100 U/ml penicillin-streptomycin (DMEM/2% FBS). The DMEM/2% FBS containing 20 μM *all-trans* retinoic acid was replaced once every two days until the cells were ready to be used in the downstream assays.

**Primary culture**

All primary culture procedures were performed following the Animal Care Guidelines of CNU. Mouse and rat primary cortical neuronal cells were isolated from the cerebral cortices of C57BL/6 mice and Sprague-Dawley rats at embryonic day 14, respectively (Koatech). These cortices were then incubated in dissection/dissociation medium [1X hank's balanced salt solution (Thermo Scientific), 1 mM sodium pyruvate, 225 ug/ml D-glucose (Thermo Scientific), and 100 mM 4-(2-hydroxyethyl)-1-piperazineethanesulfonic acid (HEPES, Thermo Scientific)] for 15 minutes with gentle inverting every five minutes and triturated to single cells using the pipetting method. The cells were seeded on multi-well plates coated with 1X poly-L-lysine (Thermo Scientific) or on coverslips (Paul Marienfield) coated with 1X poly-L-lysine. They were incubated in a plating medium [minimum essential medium with Earle’s balanced salts (MEM; WELGENE), 10 % FBS, 100 ug/ml D-glucose, 1 mM sodium pyruvate, 1X GlutaMAX (Thermo Scientific), and 100 U/ml penicillin-streptomycin] for one hour at 37 °C and 5% CO_2_ to induce attachment. The plating medium was then replaced with maintenance medium [Neurobasal medium (Thermo Scientific), 1X B-27 supplement (Thermo Scientific), 1X GlutaMAX, and 100 U/ml penicillin-streptomycin] to facilitate neuronal maturation. The maintenance medium was replaced once every two days until the cells were ready to be used in the downstream assays.

**Transfection**

siRNA and plasmid vector were used to facilitate the functional study of circTshz2-2. These constructs were transfected using Lipofectamine 3000 (Thermo Scientific) according to the manufacturer's instructions. In the case of Neuro-2A cells, 2×10^4^ cells per cm^2^ were seeded into multi-well plates and then differentiated in the DMEM/2% FBS containing 20 μM *all-trans* retinoic acid a day after seeding. On day 3 of differentiation, siRNA (Bioneer) of a final concentration of 30 nM and 500 ng of plasmid vector were transfected, respectively. The transfected cells were incubated for six hours. The medium was replaced with the DMEM/2% FBS containing 20 μM *all-trans* retinoic acid and incubated for an additional 42 hours. For the siRNA transfection of undifferentiated Neuro-2A cells, the cells were incubated in the DMEM/2% FBS for 24 hours, followed by siRNA transfection at a final concentration of 30 nM. Transfected cells were incubated for an additional six hours and then the medium was replaced with the DMEM/2% FBS and the cells were cultivated for another 42 hours.

For transfection into primary neurons, 1.5×10^5^ cells per cm^2^ were seeded into multi-well plates containing a poly-l-lysine coated coverslip, and then when the cells reached five days *in vitro* (DIV 5) they were transfected with target siRNAs at a final concentration of 30 nM. Transfected cells were incubated for six hours and then the medium was replaced with a maintenance medium and incubated for an additional 42 hours.

To analyze the primary neuron's structure after circTshz2-2 knockdown, the pMAX-GFP plasmid (Lonza) was transfected using FuGENE 6 (Promega) according to the manufacturer's instructions. When the circTshz2-2-depleted cells reached DIV 6, 1 μg pMAX-GFP plasmid was transfected into the cells and incubated for six hours. Then this medium was replaced with a maintenance medium and incubated for an additional 18 hours.

**siRNA design**

siRNAs targeting the back-splicing junction of circTshz2-2 were designed as previously described ^1^. Briefly, siDESIGN Center (https://horizondiscovery.com/en/products/tools/siDESIGN-Center) and i-Score Designer (https://www.med.nagoya-u.ac.jp/neurogenetics/i_Score/i_score.html) were used to identify siRNAs targeting the black splice junction of circTshz2-2. The circTshz2-2 siRNAs and the AccuTarget negative control siRNA were then synthesized by Bioneer and the sequences of the siRNAs used in these assays are listed in Supplementary Table S1.

**Cloning of circRNA**

To construct circTshz2-2 overexpression vector, sequences of circTshz2-2 were amplified using the primer set containing restriction enzyme sites (*PacI* and *SacII*, respectively). The PCR product was inserted into pcDNA3.1 (+) Laccase2 MCS Exon Vector, which is a gift from Jeremy Wilusz (Addgene plasmid #69893), using EZ-Fusion Cloning Kit (Enzynomics) according to the manufacturer's instructions. The primer sequences are listed in Supplementary Table S2.

**Cell fractionation**

Undifferentiated and differentiated Neuro-2A cells were separated into nuclear and cytoplasmic fractions as follows. First, the cells were collected and treated with buffer A [10 mM HEPES (pH 7.9, Thermo Scientific), 10 mM KCl (Sigma Aldrich), 0.1 mM EDTA (Sigma Aldrich), 1 mM DTT (Sigma Aldrich)] and then fractionated as previously reported ^1^. Briefly, after incubation on ice for 25 minutes, cell suspensions were supplemented with 10% Nonidet P-40 (NP-40, Thermo Scientific) to a final concentration of 0.25% and incubated for an additional two minutes. These cells were then centrifuged and the cytoplasmic RNA was isolated from the supernatant using TRIzol LS reagent (Invitrogen). The pellet was resuspended in K100 buffer D [20mM Tris (pH 8.0, Thermo Scientific), 100mM KCl, 0.2mM EDTA] and centrifuged to produce the nuclear RNA fraction. Precursor glyceraldehyde 3-phosphate dehydrogenase (pre-Gapdh) mRNA was used as a control in the nuclear fraction, and mature Gapdh mRNA was used as the cytoplasmic control.

**RNA-sequencing**

Total RNA was isolated from negative control and circTshz2-2-depleted differentiated Neuro-2A cells at day 5 using TRIzol Reagent (Thermo Fisher Scientific) according to the manufacturer's instructions. DNase I (Takara) was then added and incubated for an hour to remove residual DNA and the RNA was quantified using an ND-1000 spectrophotometer (Thermo Scientific). RNA integrity was confirmed using the 2100 Expert Bioanalyzer (Agilent) and the Ribo-Zero Gold rRNA Removal Kit (Illumina) was used to remove rRNA. The TruSeq Stranded Total RNA Kit (Illumina) was then used to construct the RNA sequencing library which was then sequenced by a HiSeq 2500 (Illumina) in the paired-end mode with 100 sequencing cycles.

We then used two previously described approaches to select those genes that experience statistically significant changes in expression following treatment with circTshz2-2 siRNAs ^2^. In brief, FASTQ reads were filtered and trimmed with Trimmomatic ^3^ and then applied to either method. In the first approach, the filtered reads were aligned to the mouse genome (mm10) using STAR ^4^, and Cuffnorm was used to calculate Fragments Per Kilobase of transcript per Million mapped reads (FPKM) ^5^. An unpaired two-tailed t-test was then used to select any significantly changed genes. In the second approach, the filtered reads were used for transcript quantification by Salmon quantifier ^6^, and edgeR was used to calculate the *p*-value of expression change ^7^. We then selected the significantly changed genes from each approach where the average *p*-value from both evaluations was less than 0.05. These genes were then filtered again to obtain those genes with log2 changes in expression greater than 0.5 or less than -0.5.

**Bioinformatics analysis**

The genomic information used in this study was obtained from the mouse (GRCm38/mm10) and human (GRCh37/hg19) genome data in the UCSC Genome Browser (https://genome.ucsc.edu/). The YY1 binding sequences were identified using the public data from ENCODE Candidate Cis-Regulatory Elements (cCREs); *Bdnf* promoter-like signature (PLS) of exon 4 (Accession: EM10E0705371) and exon 6 (Accession: EM10E0705372). The tissue distribution of human TSHZ2 was obtained from the NIH Genotype-Tissue Expression (GTEx) project (Release V8) (https://gtexportal.org/). The expression levels of circTshz2-2 during mouse hippocampal development and adult brain development were analyzed using public RNA-seq data from the Gene Expression Omnibus (GEO) dataset (Accession: GSE61991) ^8^.

Gene Ontology Resource (http://geneontology.org/) was used for gene ontology (GO) analysis of selected genes from the RNA-seq data. Of the 200 genes differentially expressed in response to circTshz2-2 knockdown, 169 were protein-coding genes which were then analyzed by selecting their "biological process" terms. The gene list was then evaluated using the PANTHER classification system and Fisher's exact test followed by a calculation of the false discovery rate (FDR). The top 10 list was selected in order of significance determined by their FDR and included in Supplementary Table S3.

We used both the BART (http://bartweb.org/) and the ChEA3 (https://maayanlab.cloud/chea3/) systems to identify the transcription factors and chromatin regulators involved in circTshz2-2-mediated *Bdnf* regulation. The list of genes identified from the RNA-seq data as differentially expressed in response to circTshz2-2 knockdown was then used as the input for the BART tool, and the top 10 factors were selected based on their Irwin-Hall *p*-value. The same list was used in the ChEA3 tool and the top 10 factors based on their Integrated Scale Rank were selected. These transcription factors are listed in Supplementary Table S3.

We then used RPIseq (http://pridb.gdcb.iastate.edu/RPISeq/) to predict the interaction probability between circTshz2-2 and specific transcriptional regulators ^9^. The circTshz2-2 and transcription regulator sequences were used as input, and the score (range from 0 to 1) of the interaction probabilities, predicted using random forest (RF) and support vector machine (SVM) classifiers, were used as a value to draw graphs in the Figure. These sequences, information, and predicted interaction scores are listed in Supplementary Table S3.

**RNA isolation and PCR**

Total RNA was isolated using TRIzol Reagent (Ambion) according to the manufacturer's instructions. This RNA was quantified using a NanoPhotometer (IMPLEN) and reverse-transcribed to complementary DNA (cDNA) using random hexamers (Thermo Fisher Scientific) and RevertAid reverse transcriptase (Thermo Fisher Scientific). A semi-quantitative PCR was then performed using nTaq DNA polymerase (Thermo Scientific) in the Master cycler Nexus X2 (Eppendorf) and the results of this PCR were evaluated using 1.5 % agarose gel electrophoresis and Image J (V1.53c) provided by the National Institute of Health (NIH) ^10^. The expression of circRNA or mRNA was normalized against the expression of *Gapdh* and the primer sequences are listed in Supplementary Table S1.

Quantitative reverse transcription-PCR (qRT-PCR) was performed on the same cDNA using Power SYBR green PCR master mix (Applied Biosystems) and the Step One Plus real-time PCR system (Applied Biosystems). Gene expression was normalized against *Gapdh* and the relative expression levels were analyzed using the 2^-(ΔΔCt)^ calculation method. These primer sequences are also listed in Supplementary Table S1.

**Western blot**

The western blot analysis was conducted as previously reported ^11^. The cells and tissues were lysed in ice-cold RIPA buffer (Translab) for 10 minutes on ice and the protein extracts were then quantified using a BCA assay kit (Thermo Fisher Scientific) according to the manufacturer's instructions. Protein (15-25 μg) was electrophoresed on 8–12% sodium dodecyl sulfate (SDS)-polyacrylamide gel, which was then transferred onto polyvinylidene difluoride (PVDF; Merck Millipore) membranes in absolute methanol (Thermo Fisher Scientific). These membranes were then incubated in blocking solution [5 % bovine serum albumin (BSA; GenDEPOT) and skimmed milk (BD Bioscience) to facilitate the detection of phosphorylated and native forms of the proteins, respectively] for an hour at room temperature. These membranes were then incubated with the appropriate primary antibodies (1:1000) overnight at 4⁰C. These primary antibodies included SYP (Abcam, ab32127), PSD-95 (Abcam, ab18258), Cyclin B2 (Santa Cruz, sc-28303), p-CDK1-Y15 (Santa Cruz, sc-136014), CDK1 (Santa Cruz, sc-54), BDNF (Abcam, ab108313), and beta-actin (Cell signaling, 4970). After incubation with appropriate horseradish peroxidase (HRP) labeled secondary antibody (1:5000; Santa Cruz) for an hour at room temperature, the membranes were incubated in ECL solution (Thermo Fisher Scientific) and visualized using Fusion Solo (Vilber). Protein expression was measured using Image J software (V1.53c) provided from NIH ^10^ and normalized against beta-actin.

**Neurite length and Sholl analysis**

Neural structures were analyzed as previously reported ^11^. Selected Neuro-2A and primary neurons were analyzed using ImageJ software (V1.53c) provided from NIH ^10^ and evaluated for neuritic complexity including evaluations of neurite length, the number of neurites from each soma, the number of secondary branches, and the number of intersections between the neurite and concentric rings. The number of neurites from the soma and the number of secondary branches were only measured when their length was greater than 1 μm.

The morphology of selected primary neurons was reconstructed using a manual tracing method and over 10 neurons per group were selected and analyzed in a blinded manner. Using the Sholl analysis plugin in Image J software ^10^, the soma center was selected using the pointed tool and used to quantify size-related parameters, such as the number of intersections and total neuritic length, from radii between 10 μm and 600 μm, with a 5 μm step size. The number of samples per radius was set at three and the degree of polynomial fit was selected as 'best-fitting degree'. We then selected and combined the data from the intersection columns in the Sholl profile list.

**Cell cycle analysis**

  The Neuro-2A cells were washed with 1X PBS and fixed in 70% ice-cold ethanol (Thermo Fisher Scientific) for 30 minutes. The fixed cells were centrifuged and washed with 1X PBS three times before being treated with 50 μl RNase A (100ug/ml; Sigma Aldrich) for 30 minutes at 37 °C. These cells were then treated with 400 μl of propidium iodide (50 µg/ml PI; Sigma Aldrich) and then used for flow cytometry after incubation for 30 minutes at room temperature. The cells were analyzed using FACSDiva software and a FACSCanto II cell analyzer (BD bioscience) with excitation at 498 nm and emission at 578nm (phycoerythrin; PE). The cell cycle phase distribution was analyzed using the Watson (Pragmatic) model in FlowJo^TM^ software (v10.7.1).

**Immunofluorescence**

Immunocytochemistry was performed as previously described ^11^. Neuro-2A cells were fixed in 2 % paraformaldehyde (Sigma Aldrich) for 15 minutes and then incubated with primary beta-tubulin and gamma-tubulin antibodies (cell signaling) in gelatin blocking buffer [0.1 % gelatin (Sigma Aldrich), 0.3 % Triton X-100 (Thermo Scientific), 16 mM sodium phosphate (Sigma Aldrich), 450 mM NaCl (Sigma Aldrich) at pH 7.4] overnight at 4⁰C. Cells were then rinsed three times in 1X PBS and incubated with Alexa 488-conjugated anti-mouse and Alexa 594-conjugated anti-rabbit antibodies (Invitrogen) for two hours at room temperature. Cells were counterstained and mounted using a mounting medium containing 4´,6´-diamidino-2-phenylindole (DAPI, Thermo Scientific) for five minutes, and images were captured using an Eclipse Ts2 fluorescent microscope (Nikon).

**RNA-binding protein immunoprecipitation**

We used RNA-binding protein immunoprecipitation (RNA-IP) to determine the identity of the proteins bound to circTshz2-2. These evaluations were completed using a Magna RIP kit (Millipore) according to the manufacturer's instructions. Briefly, the cells were lysed in RIP lysis buffer containing a protease inhibitor cocktail and an RNase inhibitor and then centrifuged. The cell lysate was then incubated with antibody-conjugated magnetic beads overnight at 4⁰C with these beads treated with primary antibodies against YY1 (Santa Cruz, sc-7341), CtBP (Santa Cruz, sc-17759), CtIP (Santa Cruz, sc-271339), and SUZ12 (Santa Cruz, sc-271325). The immunoprecipitates were then incubated in proteinase K buffer containing 10% SDS at 55⁰C for 30 minutes while shaking to digest the protein and the RNAs were extracted using phenol: chloroform: isoamyl alcohol (125:24:1, Thermo Fisher Scientific) and chloroform (Thermo Fisher Scientific), and precipitated with ethanol. The purified RNA was reverse-transcribed and subjected to semi-quantitative PCR.

**Osmotic pump implantation**

Stereotaxic surgery and osmotic pump implantation were performed as reported previously with a slight modification ^12^. Briefly, ten-week-old male, wild-type C57BL/6J and obese C57BL/6J-*ob*/*ob* mice were anesthetized with an intraperitoneal injection of 0.3mg/g (bodyweight) 2, 2, 2-tribromoethanol/2-methyl-2-butanol (Sigma) and maintained in 1-2% isoflurane with a mixed air using anesthesia instrument and mask (Harvard Apparatus). The mice were placed on a homeothermic blanket (Harvard Apparatus) to maintain body temperature. Brain infusion kit and osmotic pump (Alzet) were filled with a 100 ul solution containing siPORT-NeoFX (Invitrogen) and two independent siRNAs for circTshz2-2 at a final concentration of 5 μM and tightly assembled. The assembly was incubated with sterile saline for 16 hours at 37⁰C and infused into the lateral ventricle (mediolateral 1.0mm, anteroposterior 0.3mm, dorsoventral 2.5mm) of the mouse brain for three days using a stereotaxic instrument (Harvard Apparatus).

**T-maze spontaneous alternation test**

The T-maze behavioral test was performed as reported previously with a slight modification ^13^. The T-maze was constructed using white plastic walls with three arms (35×9×15 cm). The mice were habituated for 5 minutes a day before the initial trial. Each mouse was placed with its head facing the dead end of the start arm and allowed to move freely in the three arms of the T-maze during a 5 minutes testing period. The entry of four paws into one arm was defined as an arm entry. The movement of each mouse was recorded using a digital camera. Each record was converted to image sequences to track mouse movement using the Image J (V1.53c) plugin, Animal Tracker ^14^. The total distance, velocity, and immobility time of each mouse were extracted as the parameters of locomotor activity. The alternation was defined as multiple entries into the three different arms on overlapping triplet sets. The percentage of spontaneous alternation was calculated; [(number of alternation) / (total arm entries – 2)] multiplied by 100.

**Statistical analysis**

Data are represented as the mean, log value, min to max with mean, or mean ± SEM depending on the histogram. Experimental samples were randomly assigned into control or experimental groups, investigators were blinded to experimental conditions, and no sample was excluded for analysis. The group sample size is typically set to be three for *in vitro* and five for *in vivo* experiments to optimize the efficiency and power of statistical tests. The normal distribution and similar variance within each comparison group of data were checked before statistical tests. An unpaired two-tailed t-test with Welch's correction and an ordinary two-way ANOVA were used to analyze comparisons between control and experimental samples and statistical significance was established when the p-value was less than 0.05.

**Reference**

1. Lim YH, Ryu J, Kook H, Kim YK. Identification of Long Noncoding RNAs Involved in Differentiation and Survival of Vascular Smooth Muscle Cells. *Mol Ther Nucleic Acids* 2020; **22:** 209-221.

2. Song J, Kim YK. Discovery and Functional Prediction of Long Non-Coding RNAs Common to Ischemic Stroke and Myocardial Infarction. *J Lipid Atheroscler* 2020; **9**(3)**:** 449-459.

3. Bolger AM, Lohse M, Usadel B. Trimmomatic: a flexible trimmer for Illumina sequence data. *Bioinformatics* 2014; **30**(15)**:** 2114-2120.

4. Dobin A, Davis CA, Schlesinger F, Drenkow J, Zaleski C, Jha S *et al.* STAR: ultrafast universal RNA-seq aligner. *Bioinformatics* 2013; **29**(1)**:** 15-21.

5. Trapnell C, Roberts A, Goff L, Pertea G, Kim D, Kelley DR *et al.* Differential gene and transcript expression analysis of RNA-seq experiments with TopHat and Cufflinks. *Nat Protoc* 2012; **7**(3)**:** 562-578.

6. Patro R, Duggal G, Love MI, Irizarry RA, Kingsford C. Salmon provides fast and bias-aware quantification of transcript expression. *Nat Methods* 2017; **14**(4)**:** 417-419.

7. Robinson MD, McCarthy DJ, Smyth GK. edgeR: a Bioconductor package for differential expression analysis of digital gene expression data. *Bioinformatics* 2010; **26**(1)**:** 139-140.

8. You X, Vlatkovic I, Babic A, Will T, Epstein I, Tushev G *et al.* Neural circular RNAs are derived from synaptic genes and regulated by development and plasticity. *Nat Neurosci* 2015; **18**(4)**:** 603-610.

9. Muppirala UK, Honavar VG, Dobbs D. Predicting RNA-protein interactions using only sequence information. *BMC Bioinformatics* 2011; **12:** 489.

10. Schindelin J, Arganda-Carreras I, Frise E, Kaynig V, Longair M, Pietzsch T *et al.* Fiji: an open-source platform for biological-image analysis. *Nat Methods* 2012; **9**(7)**:** 676-682.

11. Yoon G, Kim YK, Song J. Glucagon-like peptide-1 suppresses neuroinflammation and improves neural structure. *Pharmacol Res* 2020; **152:** 104615.

12. DeVos SL, Miller TM. Direct intraventricular delivery of drugs to the rodent central nervous system. *J Vis Exp* 2013; (75)**:** e50326.

13. Deacon RM, Rawlins JN. T-maze alternation in the rodent. *Nat Protoc* 2006; **1**(1)**:** 7-12.

14. Gulyas M, Bencsik N, Pusztai S, Liliom H, Schlett K. AnimalTracker: An ImageJ-Based Tracking API to Create a Customized Behaviour Analyser Program. *Neuroinformatics* 2016; **14**(4)**:** 479-481.
